# Estimation model for habitual 24-hour urinary-sodium excretion using simple questionnaires from normotensive Koreans

**DOI:** 10.1371/journal.pone.0192588

**Published:** 2018-02-15

**Authors:** Ji-Sook Kong, Yeon-Kyung Lee, Mi Kyung Kim, Mi-Kyeong Choi, Young-Ran Heo, Taisun Hyun, Sun Mee Kim, Eun-Soon Lyu, Se-Young Oh, Hae-Ryun Park, Moo-Yong Rhee, Hee-Kyong Ro, Mi Kyung Song

**Affiliations:** 1 Division of Cancer Epidemiology and Prevention, National Cancer Center, Goyang, Gyeonggi, South Korea; 2 Department of Food Science and Nutrition, Kyungpook National University, Daegu, South Korea; 3 Division of Food Science, Kongju National University, Yesan, South Korea; 4 Department of Food and Nutrition, Chonnam National University, Gwangju, South Korea; 5 Department of Food and Nutrition, Chungbuk National University, Cheongju, South Korea; 6 Department of Family Medicine, College of Medicine, Korea University, Seoul, South Korea; 7 Department of Food Science and Nutrition, Pukyong National University, Busan, South Korea; 8 Department of Food and Nutrition, Kyunghee University, Seoul, South Korea; 9 Department of Food and Nutrition, Myongji University, Yongin, South Korea; 10 Cardiovascular Center, Dongguk University Ilsan Hospital, Goyang, Gyeonggi, South Korea; 11 Department of Food and Nutrition, Dongshin University, Naju, South Korea; 12 Biometrics Research Branch and Biostatistics Collaboration Unit, Division of Cancer Epidemiology and Prevention, Research Institute and Hospital, National Cancer Center, Goyang, Korea, National Cancer Center, Goyang, Gyeonggi, South Korea; The University of Tokyo, JAPAN

## Abstract

This study was conducted to develop an equation for estimation of 24-h urinary-sodium excretion that can serve as an alternative to 24-h dietary recall and 24-h urine collection for normotensive Korean adults. In total, data on 640 healthy Korean adults aged 19 to 69 years from 4 regions of the country were collected as a training set. In order to externally validate the equation developed from that training set, 200 subjects were recruited independently as a validation set. Due to heterogeneity by gender, we constructed a gender-specific equation for estimation of 24-h urinary-sodium excretion by using a multivariable linear regression model and assessed the performance of the developed equation in validation set. The best model consisted of age, body weight, dietary behavior (‘eating salty food’, ‘Kimchi consumption’, ‘Korean soup or stew consumption’, ‘soy sauce or red pepper paste consumption’), and smoking status in men, and age, body weight, dietary behavior (‘salt preference’, ‘eating salty food’, ‘checking sodium content for processed foods’, ‘nut consumption’), and smoking status in women, respectively. When this model was tested in the external validation set, the mean bias between the measured and estimated 24-h urinary-sodium excretion from Bland-Altman plots was -1.92 (95% CI: -113, 110) mmol/d for men and -1.51 (95% CI: -90.6, 87.6) mmol/d for women. The cut-points of sodium intake calculated based on the equations were ≥4,000 mg/d for men and ≥3,500 mg/d for women, with 89.8 and 76.6% sensitivity and 29.3 and 64.2% specificity, respectively. In this study, a habitual 24-hour urinary-sodium-excretion-estimation model of normotensive Korean adults based on anthropometric and lifestyle factors was developed and showed feasibility for an asymptomatic population.

## Introduction

Sodium intake has been reported to be a known risk factor for hypertension in many, animal as well as epidemiological studies [1–4]. Excess sodium intake additionally is a known risk factor for cardiovascular diseases and stroke [5, 6], renal disease [7], stomach cancer [8], and osteoporosis [9, 10]. Although the WHO recommends a sodium intake of <2,000 mg/d (<5 g/d salt) to prevent diet-related chronic diseases [11], the average level among Koreans exceeds that level by approximately two-fold [12]. Sodium intake is considered a national problem, and the Korean government has implemented policies to reduce it.

Several sodium-intake estimation methods have been proposed: dietary assessments based on 24-h diet questionnaires or food-frequency questionnaires, and urinary assessments based on 24-h urine or spot urine. Dietary assessment of sodium intake has been reported to be inaccurate, due to under- or over-estimation, though it has a lower subject burden and is relatively inexpensive [13, 14]. The alternative 24-h urinary assessment has been considered to be the most valid and reliable method, and in fact is the gold standard [14] for 24-h sodium intake estimation. However, 24-h urine sampling is highly burdensome, not to mention time- and cost-intensive for large populations. In efforts to overcome these limitations, several studies have developed equations for facile as well as precise 24-h urinary-sodium excretion (24-hUNa) estimation based on spot urine and anthropometric factors [15–20]. Unfortunately, these methods are as yet not readily available to large populations, as they require laboratory analysis of the urine sample.

Currently, most models for estimation of 24-hUNa require morning fasting or spot urine sampling [15, 20–24]. A model is needed for simple and effective Na intake (or excretion) estimation for large-scale epidemiologic studies with no urine collection. However, to the authors’ best knowledge, no studies have established any 24-hUNa estimation methods that consider epidemiological risk factors. In the present study, we aimed to develop an accurate and simple equation for estimation of 24-hUNa using questionnaires, because dietary assessments based on 24-h diet questionnaires or food-frequency questionnaires are inaccurate while 24-h urine collection and the spot unirary method require lab analysis and are inconvenient.

## Materials and methods

### Study population

Study 1 (training set) for equation development was carried out between September and December 2014, and study 2 (external validation set) for validation of the developed equation was conducted from September to December 2015. For study 1, a total of 640 healthy men and women aged 20–69 years living in Korean districts within 4 areas (central, middle, southeast, and southwest) were enrolled in this study. An equal number of subjects (16 men and 16 women) were enrolled from each district and assigned to one of five 10-year age groups (20~29, 30~39, 40~49, 50~59, and 60~69 years) in order to ensure uniformity of gender and age distributions. For study 2, 200 healthy men and women aged 20–69 years living in the same 4 Korean districts as the study 1 subjects were enrolled. An equal number of subjects (5 men and 5 women) in each district were enrolled and assigned to one of the five 10-year age groups. For study 1, the 640 subjects provided comprehensive data in the following forms: dietary and lifestyle questionnaires, a 24-h dietary recall (twice), a semi-quantitative food frequency questionnaire (SQ-FFQ), a salty-taste assessment, and a 24-h urine sample (twice) (S1 Table and S1 Fig). For study 2, the 200 subjects provided comprehensive data in the forms of the above-noted dietary and lifestyle questionnaires and a 24-h urine sample (S1 Table and S1 Fig). The eligible study subjects were individually informed of the study purpose and the entire data collection procedure by trained dietitians using detailed written documents (Supporting Information Survey questionnaire). The questionnaire is provided in S1 Survey questionnaire (original language in Korean and translated in English). All of the subjects participating in the study had submitted their written informed consent. All were healthy, with no history of diagnosis of hypertension, congestive heart failure, diabetes, or kidney disease. Any eligible subjects who were on medication, pregnant or lactating, or under dietary restriction, were excluded. This study was approved by the Institutional Review Board of Kyungpook National University (IRB 2014–0053), and all of the participants provided written informed consent. There were no overlapping participants between the training and validation sets.

### Dietary measurements using questionnaire

The subjects’ dietary measurements were obtained by interviewer-administered questionnaires: a dietary behavior questionnaire, 24-h recall questionnaire (twice), the SQ-FFQ, and a salty-taste assessment. A summary of the study 1 and study 2 variables is provided in S1 Table. The dietary behavior questionnaire is comprised of 20 items regarding sodium and potassium intake. The 24-h dietary recall questionnaire obtained information on each participant’s remembered dietary intake, including all food and drink, throughout the day prior to the interview. This data subsequently was converted to actual sodium intake amounts using CAN-pro Professional 4.0 (The Korean Nutrition Society, Seoul, Korea) and the Rural Development Administration’s Food Composition Table (South Korea, 8^th^ revision). The mean of the two 24-h-recalled sodium intakes was used as the participants’ “sodium intake”. Sodium intake is measured using a modified SQ-FFQ with 78-dish items (that showed higher sodium content per portion size and higher consumption frequency) reflecting subjects’ sodium intake over the past 6 months [25–27]. In the present study, the SQ-FFQ was performed one time to calculate sodium intake in the training set. Salty-taste assessment [28, 29] was evaluated using a salty-taste assessment kit.

### Urine collection and analysis

The subjects were given written guidelines on the method of urine collection (S1 Fig). The two 24-h urine collections were scheduled at a minimum interval of 3 days. When there was a necessity to adjust the test date due to difficulties such as urine loss or the menstrual period of women participants, a minimum interval of 3 days was required between the first and second 24-h urine collections. At the start time of the collection, they were instructed to avoid the first urine in the morning (not included as part of the collection), and to begin urine collection with the following urination and to end with the first urine of the next day. The participants recorded the start and finish time and the volume of urination. The subjects were instructed to collect urine in 500 mL urine beakers at every urination and to record the quantity. Upon obtainment of the samples, 15 mL of first morning urine was stored as the spot urine sample in a 50 mL tube, and the remaining urine was combined with the 24-h urine sample. Compliance with 24-h urine collections was confirmed when the value [collected urine volume/ (body weight × 21)] was over 0.7 [30]. When the value was lower than 0.7 and when the creatinine concentration of 24-h urine was lower than the reference (value estimated based on sex, age, and weight of subject), the data were excluded from the analysis. After the two samples were confirmed, the respective two sodium excretion values were averaged, which value, as adjusted for individual-level and day-to-day variations in sodium excretion, was used as the representative one [1, 31]. For the training set (study 2), non-consecutive 24-h urine collections were conducted during the study period, scheduled according to subject convenience (S1 Fig). The collected urine samples, maintained under refrigeration during the collection period, were brought in iceboxes to the research laboratory (Green Cross Labcell, Yongin, South Korea), where sodium (Na+), potassium (K+) and creatinine (Cr) analyses were carried out. The spot and 24-h urinary-sodium and potassium concentrations were analyzed by the Ion Selective Electrode (ISE) method, and 24-h urinary creatinine by the Kinetic Colorimetry Assay method. Meanwhile, the 24-hUNa were calculated by the following equation: 24-hUNa (mmol/day) = concentration of 24-hUNa (mmol/L) * 24-h urine volume (L/day). We did not use any criteria to evaluate the appropriateness of the spot urine samples (e.g. urine creatinine concentration).

### Other measurements

The subjects’ socio-demographic characteristics and several lifestyle factors were collected by an interviewer-administered questionnaire. These variables included age, region (residence), smoking status, regular exercise, education, occupation, and household income. The regions were classified into Seoul and Gyeonggi province (central district), Chungcheong province (middle district), Gyeongsang province (southeast district), and Jeolla province (southwest district). Smoking status was classified as non-smoker, past smoker, or current smoker. Regular exercise was defined as more than 1 time per week and 30 min per time. Education was classified into high school graduate or less and college or more. Household income (units of ten thousand won) was classified into 200 or less, 200~300, 300~400, and 400 or more. Anthropometric measurements were obtained by well-trained interviewers and included body weight, height, waist circumference, hip circumference, and blood pressure. The subjects’ height and weight (without shoes) were obtained by standardized techniques and calibrated equipment. The body-mass index (BMI) formula was as follows: weight (kg) / height (m) ^2^.

### Development of equation for estimation of 24-hUNa

The potential risk factors considered in the prior analysis were dietary behavior factors, anthropometric measurements, and lifestyle factors, as determined by dietary questionnaires (dietary behavior questionnaire, 24-h recall questionnaire, SQ-FFQ, salty-taste assessment), socio-demographic characteristics (age, region, education, occupation, household income) and two lifestyle factors (smoking, regular exercise), and anthropometric measurements (body weight, height, waist circumference, hip circumference, blood pressure). We took two steps to develop an equation estimating 24-hUNa in a training set (n 640; 320 men and 320 women). The first step is the process of selecting meaningful candidate variables, based on the following three criteria: significant (*P*<0.2) factors from the univariate models, important variables in the previous studies regardless of significance [32], and variables with a <2.0 variance inflation factor (VIF) indicating minimal collinearity (S2 Table). In the second step, stepwise selection with the exclusion criterion at *P*≥0.15 was performed to determine the 24-hUNa equation. After these two steps, we adopted the final equations for each gender.

### Statistical analysis

Data on the baseline characteristics of the study subjects are presented as the mean and standard deviations for continuous variables and, for categorical variables, as the frequency (with percentage). The continuous and categorical variables were tested using Student’s t-test and chi-square test to assess the difference between data sets and between genders, respectively. As Korean sodium excretions have significantly differed by gender in previous studies [33–35] as well as our present study (S2 Fig, 174 mmol/d in men and 136 mmol/d in women, *P* <0.001, Wilcoxon rank-sum test), all of the analyses were performed separately for each gender. Significant (*P*<0.2) factors from the univariate models as well as non-significant but valuable factors were considered, and stepwise selection was used to determine the final models. The performances of the models were investigated according to Spearman’s correlation coefficient (r) and the mean bias with 95% limits of agreement. Spearman’s correlation coefficient was used to evaluate the relationship of the measured 24-hUNa with the estimated 24-hUNa or other sodium-related variables, and was visualized in a scatter plot. The mean bias between the measured and estimated 24-hUNa was used to explore similarity, and we also visualized it in a Bland-Altman plot with 95% limits of agreement [36]. Also, the difference values between the measured and estimated 24-hUNa were summarized as the median, range (minimum, maximum) and inter-quartile range (IQR). Joint classification analysis was used to describe the agreement between the measured and estimated 24-hUNa. For this analysis, the subjects were divided into quartile groups according to their measured and estimated 24-hUNa, and same, adjacent, and opposite quartile classifications were defined. Based on the previous studies, the estimate of sodium intake was confirmed to be 86% of 24-hUNa [37, 38]. When applying this criterion to the estimated 24-hUNa derived from the proposed equation, the cut-points in our data were ≥131 mmol/d (equivalent to a sodium intake of 3,500 mg/d) among women and ≥150 mmol/d (equivalent to a sodium intake of 4,000 mg/d) among men. All statistical tests adopted in this study were two-tailed ones at significant level of 0.05. The analyses were performed using Statistical Analysis System (version 9.3, SAS Institute, Inc., Cary, NC) and R statistical software (version 3.3.2).

## Results

### Subjects’ characteristics

The subjects’ characteristics in the training and validation sets are shown in **Table 1**. The number of subjects was exactly balanced in terms of (10-year) age group, gender, and home region. Intra-gender, there were no significant differences in anthropometric or demographic data, lifestyle factors or dietary and urinary-sodium levels between the training and validation sets, with the exception of education level. However, there were significant inter-gender differences in the anthropometric (e.g., weight, height, BP, etc.), demographic (e.g., Education status, etc.), lifestyle, dietary (by 24-h recall method and by SQ-FFQ method) and urinary-sodium-level variables (Table 1 summarizes the results as analyzed by t-test or chi-square test). Subsequent gender-subgroup analyses were performed for food preferences, food choices, eating patterns and nutrition intakes considered to affect sodium excretion by gender [39–41]. Although our lifestyle and dietary questionnaires were the same for the two genders, the selected variables in the estimated model differed by gender. The models thus developed were each validated in an independent validation set.

**Table 1:** General characteristics of study subjects.

	Men			Women			*P*^c^	*P*^d^
Variables	Training set (n, 320)^a^	Validation set (*n*, 100)	*P*^b^	Training set (n, 320) a	Validation set (*n*, 100)	*P*^b^		
Age (yrs)	43.7 ± 14.3	44.5 ± 13.9	0.6411	43.7 ± 14.6	44.4 ± 14.4	0.6749	0.9935	0.9721
19–29 yrs, n (%)	64 (20)	20 (20)	-	64 (20)	20 (20)	-	-	-
30–39 yrs	64 (20)	20 (20)		64 (20)	20 (20)			
40–49 yrs	64 (20)	20 (20)		64 (20)	20 (20)			
50–59 yrs	64 (20)	20 (20)		64 (20)	20 (20)			
60–69 yrse	64 (20)	20 (20)		64 (20)	20 (20)			
Regions, n (%)								
Seoul and Gyeonggi province (Central district)	80 (25)	25 (25)	-	80 (25)	25 (25)	-	-	-
Chungcheong province (Middle district)	80 (25)	25 (25)		80 (25)	25 (25)			
Gyeongsang province (Southeast district)	80 (25)	25 (25)		80 (25)	25 (25)			
Jeolla province (Southwest district)	80 (25)	25 (25)		80 (25)	25 (25)			
Weight (kg)	71.4 ± 9.64	70.7 ± 9.94	0.5262	56.5 ± 7.78	55.6 ± 7.82	0.3020	<0.0001	<0.0001
Height (cm)	171 ± 5.77	171 ± 5.59	0.9913	159 ± 5.76	159 ± 5.01	0.6323	<0.0001	<0.0001
BMI (kg/m^2^)	24.3 ± 2.96	24.0 ± 2.78	0.4083	22.3 ± 2.93	22.1 ± 3.25	0.4662	<0.0001	<0.0001
SBP (mmHg)	126 ± 9.25	123 ± 10.2	0.0085	118 ± 11.4	116 ± 12.0	0.1362	<0.0001	<0.0001
DBP (mmHg)	80.2 ± 7.11	80.4 ± 7.43	0.8094	75.8 ± 7.82	74.1 ± 7.93	0.0515	<0.0001	<0.0001
Smoking status, n (%)								
Non-smoker	145 (45.3)	38 (38.0)	0.4336	306 (95.6)	94 (94.0)	0.3849	<0.0001	<0.0001
Past smoker	86 (26.9)	31 (31.0)		8 (2.50)	5 (5.00)			
Smoker	89 (27.8)	31 (31.0)		6 (1.88)	1 (1.00)			
Regular exercise^e^	176 (55.0)	70 (70.0)	0.0110	163 (50.9)	56 (56.0)	0.4413	0.3419	0.0569
Education status, n (%)								
≤ High school graduate	132 (41.2)	31 (31.0)	< .0001	154 (48.1)	52 (52.0)	0.0151	<0.0001	0.0019
≥ College	188 (58.8)	69 (69.0)		166 (51.9)	48 (48.0)			
Household income (ten thousand won, n (%))								
≤ 200	135 (42.2)	44 (44.0)	0.0406	211 (66.0)	75 (75.0)	0.1030	<0.0001	0.0002
200~300	67 (20.9)	16 (16.0)		55 (17.2)	13 (13.0)			
300~400	46 (14.4)	23 (23.0)		20 (6.25)	6 (6.00)			
≥ 400	72 (22.5)	13 (13.0)		34 (10.6)	4 (4.00)			
24-h urine collection								
Sodium excretion (mmol/d)	174 ± 65.6	172 ± 60.6	0.8355	136 ± 49.8	132 ± 50.6	0.5127	<0.0001	<0.0001
Potassium excretion (mmol/d)	56.9 ± 20.6	56.8 ± 28.7	0.9611	54.1 ± 20.6	52.3 ± 18.6	0.4398	0.0830	0.1945
Creatinine excretion (g/d),	1.52 ± 0.43	1.52 ± 0.45	0.8879	0.90 ± 0.24	0.94 ± 0.19	0.1232	<0.0001	<0.0001
Volume (ml/d)	1786 ± 557	1881 ± 717	0.2257	1659 ± 509	1608 ± 616	0.4494	0.0027	0.0042
Estimated 24-h Sodium excretion^f^	174 ± 28.4	174 ± 27.1	0.9051	136 ± 20.6	134 ± 20.9	0.3437	<0.0001	<0.0001
Dietary Sodium intake								
Sodium intake by 24-h recall method (mg/d)	4071 ± 1539	-	-	3440 ± 1433	-	-	<0.0001	-
Sodium intake by SQ-FFQ method (mg/d)	3924 ± 3077	-	-	3310 ± 2528	-	-	0.00600	-

BMI, body-mass index; SBP, systolic blood pressure; DBP, diastolic blood pressure; SQ-FFQ, semi-quantitative food frequency questionnaire

a Values are shown as Mean and SD, or N (%).

b Results were analyzed by t-test or chi-square test (between Training set and Validation set).

c Results were analyzed by t-test or chi-square test in the Training set (between men and women).

d Results were analyzed by t-test or chi-square test in the Validation set (between men and women).

e Regular exercise was defined as more than 1 time per week and 30 min per time.

f Estimated 24-h urinary Na excretion (24-hUNa) using the developed equation (mmol/d)

### Development of equations

We aimed to develop an estimation model for habitual 24-hour urinary sodium excretion using a simple questionnaire. Based on stepwise multiple regression analysis, we developed gender-specific equations for estimation of 24-hUNa (**Table 2**). For men, the 7 variables incorporated into the final equation model were age, body weight, dietary behavior (‘eating salty food’, ‘Kimchi consumption’, ‘Korean soup or stew consumption’, ‘soy sauce or red pepper paste consumption’), and smoking status; for women, the 7 variables were age, body weight, dietary behavior (‘salt preference’, ‘eating salty food’, ‘checking sodium content for processed foods’, ‘nut consumption’), and smoking status (S3 Table).

**Table 2:** Equation for estimation of 24-hUNa (mmol/d), using β-coefficient estimates in multiple linear regression.

Men	= -472+(0.83*age)+(145*Body_wt)+(-12.2*M1_1)+(5.4*M1_2)+(-28.8*M2)+(11.9*M3) +(-7.26*M4)+(-12.9*M5)					
Item	Parameters	β	t-value	SE	*P*-value
	(Constant)	-472	-4.28	110	< .0001
	Age (year)	0.83	3.27	0.25	0.0012
	Body weight (kg, logarithmic transformation)	145	5.7	25.5	< .0001
M1_1	Eating salty food? Not salty	-12.2	-1.25	9.77	0.2122
M1_2	Eating salty food? Salty	5.41	0.67	8.02	0.5007
M2	Kimchi consumption, < 1/day	-28.8	-2.99	9.63	0.003
M3	Korean soup or stew consumption, ≥ 2/day	11.9	1.5	7.96	0.1347
M4	Soy sauce or red pepper paste consumption, Occasionally	-7.26	-1.02	7.15	0.3104
M5	Past smoker	-12.9	-1.61	8.01	0.1093
Women	= -321+(0.52*age)+(105*Body_wt)+(18.2*F1)+(14.2*F2)+(2.12*F3)+(-15.7*F4) +(-19.0*F5)				
Item	Parameters	β	t-value	SE	*P*-value
	(Constant)	-321	-4.07	79	< .0001
	Age (year)	0.52	2.72	0.19	0.0068
	Body weight (kg, logarithmic transformation)	106	5.35	19.8	< .0001
F1	Salt preference, Very much like	18.2	2.89	6.3	0.0041
F2	Eating salty food? Salty	14.2	2.15	6.63	0.0325
F3	Checking Na content for processed foods, No	2.12	0.35	6.01	0.7241
F4	Nut consumption, Intermediate	-15.7	-2.96	5.29	0.0033
F5	Past smoker	-19.0	-1.15	16.5	0.2494

β, beta coefficients; SE, standard error; 24-hUNa, 24-h urinary-sodium excretion

### Validation of equations for 24-h Na

#### Correlations between measured and estimated 24-hUNa

**Fig 1:**
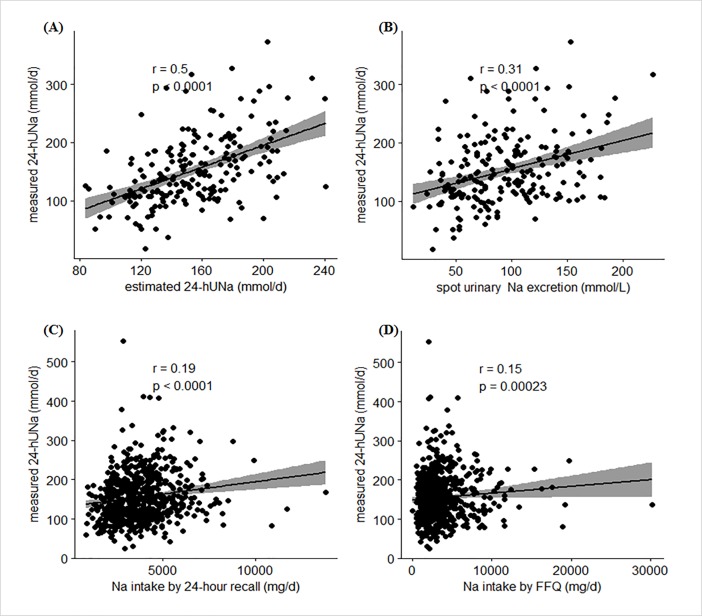
Correlation^a^ between measured 24-hUNa^b^ and other sodium-related variables (training set, study 1 & validation set, study 2) 24-hUNa, 24-h urinary-sodium excretion. a Spearman’s correlation coefficient between measured 24-hUNa and other sodium-related variables.b Measured 24-hUNa using 24-h urine (mmol/d). Panel (A): Estimated 24-hUNa (mmol/d), validation set; Panel (B): Spot urinary Na excretion (mmol/L), validation set; Panel (C): Na intake by 24-h recall (mg/d), training set; Panel (D): Na intake by FFQ (mg/d), training set.

Correlation analysis was performed to determine the correlation between the measured and estimated 24-hUNa using the developed equation (Fig 1A). Our estimated 24-hUNa using the developed equation was moderately correlated with the measured 24-hUNa (*r* = 0.5, *P<*0.0001). A Spearman’s correlation analysis of the gender subgroups showed that the correlation coefficients between the measured and estimated 24-hUNa levels were 0.35 (*P<*0.001) for men and 0.47 (*P<*0.0001) for women (S3 Fig) in the external validation set.

#### Comparison correlation with other methods

Correlation analysis was performed to determine which of the developed equations and other sodium-related variables were better correlated with the measured 24-hUNa (Fig 1). The correlations between the measured 24-hUNa and dietary Na (Fig 1C by 24-h recall and Fig 1D by FFQ) were very weak (*r* = 0.19 by 24-h recall and 0.15 by FFQ); even those between the measured 24-hUNa and spot UNa were weak (*r* = 0.31). Compared with the very weak correlations of these Na-related variables, our estimated 24-hUNa was moderately correlated with the measured 24-hUNa (*r* = 0.5). The correlation coefficient between the measured 24-hUNa and dietary sodium intake by 24-h recall was 0.19 (*P*<0.0001), which was statistically significant only among men.

#### Differences between measured and estimated 24-hUNa

**Fig 2:**
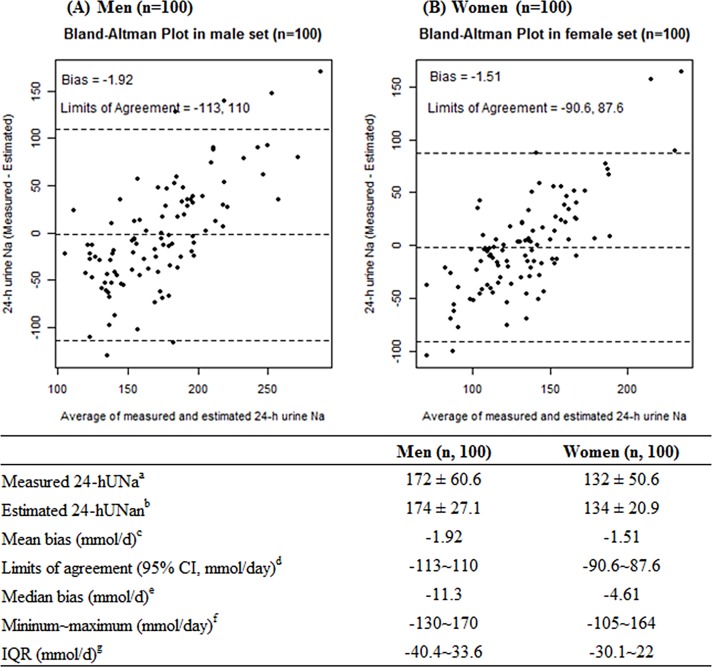
Bland-Altman plot of measured and estimated 24-hUNa by gender (validation set, study 2). The lines represent the mean bias (measured 24-hUNa—estimated 24-hUNa) and the 95% limits of agreement of the mean bias ± 1.96 × SD (the upper limit of agreement equals the mean bias ± 1.96 × SD of the difference; the lower limit equals the mean bias−1.96 × SD) (panel A, men; panel B, women). 24-hUNa, 24-h urinary-sodium excretion. ^a^ Measured 24-hUNa using 24-h urine (mmol/d). ^b^ Estimated 24-hUNa using equation (mmol/d). ^c^ Mean bias (measured 24-hUNa—estimated 24-hUNa). ^d^ Limits of agreement (measured 24-hUNa—estimated 24-hUNa): 95% upper limit-95% lower limit. ^e^ Median bias (measured 24-hUNa—estimated 24-hUNa). ^f^ Range of difference (measured 24-hUNa—estimated 24-hUNa): minimum~maximum. ^g^ IQR (Interquartile range) of difference (measured 24-hUNa—estimated 24-hUNa).

Since a smaller difference between the measured and estimated 24-hUNa indicates a better performance of the proposed equation, we calculated the mean bias using the differences between those two values. The measured and estimated 24-hUNa of the subjects are plotted in **Fig 2**. In the validation set, the mean measured and estimated 24-hUNa were 172 mmol/d and 174 mmol/d in men, and 132 mmol/d and 134 mmol/d in women, respectively. By Bland-Altman analysis, the mean biases (= measured 24-hUNa–estimated 24-UNa) between the measured and estimated 24-hUNa were -1.92 mmol/d for men and -1.51 mmol/d for women, respectively, in the validation set. The joint classification into quartiles (the same and adjacent quartiles) showed mostly good agreement between the measured and estimated 24-hUNa (77.0% for men and 82.0% for women) (**Table 3**).The equations also identified persons with sodium intake ≥3,500 mg/d in women and ≥4,000 mg/d in men (24-hUNa, 131 mmol/d and 150 mmol/d, respectively), with a sensitivity of 89.8% in men and 76.6% in women, respectively. The equations identified persons with sodium intake ≥ 2,000 mg/d (24-hUNa, 74 mmol/d) with a sensitivity of 100% (data not shown). However, after joint classification into quartiles, 5.00 and 6.00% of the men and women subjects, respectively, were misclassified.

**Table 3:** Estimation accuracy between measured 24-hUNa^a^ and estimated 24-hUNa^b^ (validation set, study 2).

Validation set	Agreement^c^			Sensitivity (≥150, 131 mmol)^d^	Specificity (≥150, 131 mmol)
	Same quartiles (n, %)	Up to adjacent quartiles (n, %)	Opposite quartiles (n, %)		
Total (*n*, 200)	66 (33.0)	159 (79.5)	11 (5.50)	84.0%	48.9%
Men (*n*, 100)	32 (32.0)	77 (77.0)	5 (5.00)	89.8%	29.3%
Women (*n*, 100)	34 (34.0)	82 (82.0)	6 (6.00)	76.6%	64.2%

24-hUNa, 24-h urinary-sodium excretion

a Measured 24-hUNa using 24-h urine (mmol/d)

b Estimated 24-hUNa using equation (mmol/d)

c Percent of subjects classified into same group and up to adjacent quartiles of estimated 24-hUNa

d Estimation accuracy between measured 24-hUNa and estimated 24-hUNa (men: ≥150 mmol, 4,000 mg, women: ≥131 mmol, 3,500 mg)

## Discussion

The gold standard for estimation of sodium excretion or intake has been considered to be the 24-h urine collection method [14], which, not coincidentally, is the most valid and reliable. However, 24-h urine sampling imposes a high burden on subjects, and is also time- and cost-intensive in a large population. In this study, we developed alternative easy, simple, low-cost and gender-specific equations for estimation of 24-hUNa. The equations utilize a simple questionnaire that includes 7 variables for men (age, body weight, four dietary variables [‘eating salty food’, ‘Kimchi consumption’, ‘Korean soup or stew consumption’, ‘soy sauce or red pepper paste consumption’] and smoking status) and 7 variables for women (age, body weight, four dietary variables [‘salt preference’, ‘eating salty food’, ‘checking sodium content for processed foods’, ‘nut consumption’] and smoking status). Each equation’s validity was evaluated with an independent dataset.

The 24-h dietary recall method has been shown to be unreliable as a sodium intake estimation method, as it typically under- or overestimates intake [42–44]. Similarly, in our study, the estimated sodium intake by 24-h recall was only weakly correlated with the measured 24-hUNa for the total subjects (*r* = 0.19, *P*<0.0001), as was the estimated sodium intake by SQ-FFQ (*r* = 0.15, *P =* 0.00023). Using the developed equations, the correlation of estimated 24-hUNa with measured 24-hUNa, in this study, was clear (*r* = 0.5, *P<*0.0001), which result is comparable with that for other sodium-related variables (spot UNa, 24-h recall, and SQ-FFQ). In a previous Korean study, the reported correlations between the measured and estimated 24-hUNa using spot urine by Kawasaki's equation, Tanaka's equation, and INTERSALT’s equation were 0.62, 0.57, and 0.58, respectively; and in a Bland-Altman analysis, the mean biases of those three equations were -30.2 mmol/d (Kawasaki's), 12.2 mmol/d (Tanaka’s), and 33.6 mmol/d (INTERSALT) [20]. In the present study, the mean bias between the measured and estimated 24-hUNa using the equation was -1.71 mmol/d. Also, the estimated 24-hUNa by each equation (Kawasaki, Tanaka, and INTERSALT) in that other Korean population [20] significantly differed from the measured 24-hUNa by paired t-test. However, in the present study, the mean differences between the measured and estimated 24-h sodium by our developed gender-specific equations were insignificant (S4 Fig). Relative to our results, a recent Chinese study [45] reported low correlations between measured and estimated 24-hUNa using spot urine by Kawasaki's equation (*r* = 0.187), Tanaka's equation (*r* = 0.293), and INTERSALT’s equation (*r* = 0.187). Moreover, they reported relatively larger mean biases: -32.2 mmol/d (-740.5 mg/d, Kawasaki's), -100.2 mmol/d (-2305 mg/d, Tanaka’s), and -121.6 mmol/d (-2797 mg/d, INTERSALT).

Our study has some limitations. The first is possible selection bias [46], as the subjects, all volunteers, might be more interested in health than subjects randomly sampled. Furthermore, our study population has a higher socioeconomic index and a relatively lower sodium intake than the more representative Korean sample utilized in a previous study [35]. As for the second limitation, our equation, though likely to have a significant influence on 24-hUNa estimation, was developed for the Korean population, and cannot be generalized to other populations [25–27]. Third, whereas our equation can be useful for prediction of 24-hUNa at the Korean population level (because the mean bias [absolute levels] was small and objectively assessed in the external validation set), our results are somewhat limited for prediction at the individual level, because of the wide limits of agreement and because of a tendency to negative bias for lower sodium excretion levels and positive bias for higher sodium excretion levels. The bias issue has been confirmed in all predictive equations (INTERSALT, Tanaka, Kawasaki, and Mage) that estimate the 24-hUNa using spot UNa [20, 41]. However, the linearity problem of this mean bias could not be solved, not even by log-transforming. Further study will be needed to resolve this issue.

Nevertheless, our study has strengths. First, we measured 24-h urine collection twice as a reference for sodium intake estimation (the gold standard); this was required for accurate measurement, because salt intake largely differs on a daily basis. Second, only a normal population was included in this study, whose sodium status was affected only by their own diet and lifestyle; in other words, we excluded subjects with chronic diseases, such as hypertension and diabetes, that might affect the sodium excretion by dietary therapy or medicinal intervention. Third, although randomized sampling was not used, gender, age, and region were evenly distributed to minimize bias (this study was conducted in 8 study centers and four regions). Fourth, our equation for urinary sodium can reflect the habitual dietary sodium intake by utilizing two urine collections adjusted for individual-level and day-to-day variability [34], while equations using spot urine and measured 24-h urinary sodium reflect the sodium level only for a specific day [16]. Accordingly, the factors used in our equation to replace the measured 24-hUNa were questionnaires for habitual sodium intake rather than for specific one-day sodium intake. Fifth, whereas other methods using equations still require laboratory analysis of urine samples, our simple-questionnaire-based approach does not. Sixth and finally, our developed formula was validated against an independent set of an additional 200 samples collected. In the meantime, we look forward to improving the accuracy of sodium excretion estimation through extensive additional research.

## Conclusions

We established an easy, low-cost, and gender-specific method for estimation of 24-hUNa using daily-habits questionnaire that accounts for age, body weight, dietary behavior, and smoking. This study enrolled healthy volunteers with normal blood pressure and no other diseases. Our habitual 24-hour urinary-sodium-excretion-estimation model of normotensive Korean adults based on anthropometric and lifestyle factors showed good performance for an asymptomatic population. This suggests that it is a feasible potential model for monitoring of sodium intake in a Korean population. Further study of the prediction model using simple questionnaires yielding the most accurate estimation of 24-h urinary-sodium excretion at both the population-mean and individual levels would be recommended.

## Supporting information

S1 Fig Study design: Training and validation sets. SQ-FFQ, semi-quantitative food frequency questionnaire.
(TIF)

S2 Fig Box-plot diagram of measured 24-hUNa distribution by gender (training set, study 1). The 24-hUNa significantly differed between men and women (*P*<0.001, Wilcoxon rank-sum test).
(TIF)

S3 Fig Correlation^a^ between measured^b^ and estimated^c^ 24-hUNa by gender (validation set, study 2). a Spearman’s correlation coefficient between measured 24-hUNa and other sodium-related variables
b Measured 24-hUNa using 24-h urine (mmol/d)
c Estimated 24-hUNa using equation (mmol/d)
Panel (A): Estimated 24-hUNa (mmol/d), men; Panel (B): Estimated 24-hUNa (mmol/d), women.
(TIF)

S4 Fig Box-plot diagram of measured 24-hUNa distribution by gender (validation set, study 2). The mean differences between the measured and estimated 24-hUNa by our developed gender-specific equations were insignificant by paired t-test.
(TIF)

S1 Table Summary of survey variables in training set (study 1) and validation set (study 2). (DOCX)

S2 Table Univariate linear regression analysis for estimation of 24-hUNa (mmol/d), using β-coefficient estimates. (DOCX)

S3 Table Gender-specific regression equations estimating 24-hUNa from age, weight, smoking status, and simple dietary questionnaire items. (DOCX)

S1 Survey Questionnaire Contains the survey questionnaire used in this study. (DOCX)
