# Supplementary figures and images for: Estimation model for habitual 24-hour urinary-sodium excretion using simple questionnaires from normotensive Koreans
Source: PLoS One. 2018 Feb 15;13(2):e0192588. doi: 10.1371/journal.pone.0192588 (PMC5813954; doi:10.1371/journal.pone.0192588)

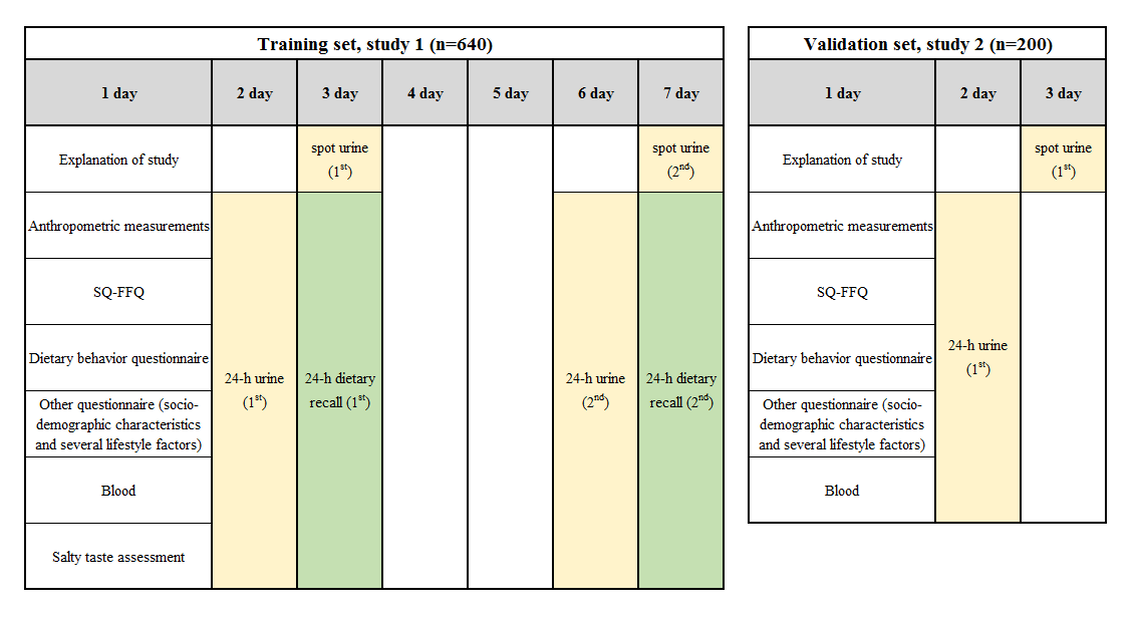

Supplement: S1 Fig — SQ-FFQ, semi-quantitative food frequency questionnaire. (TIF) [file pone.0192588.s001.tif]

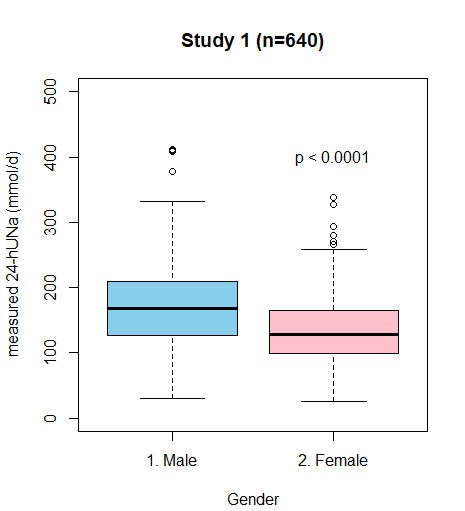

Supplement: S2 Fig — The 24-hUNa significantly differed between men and women (P<0.001, Wilcoxon rank-sum test). (TIF) [file pone.0192588.s002.tif]

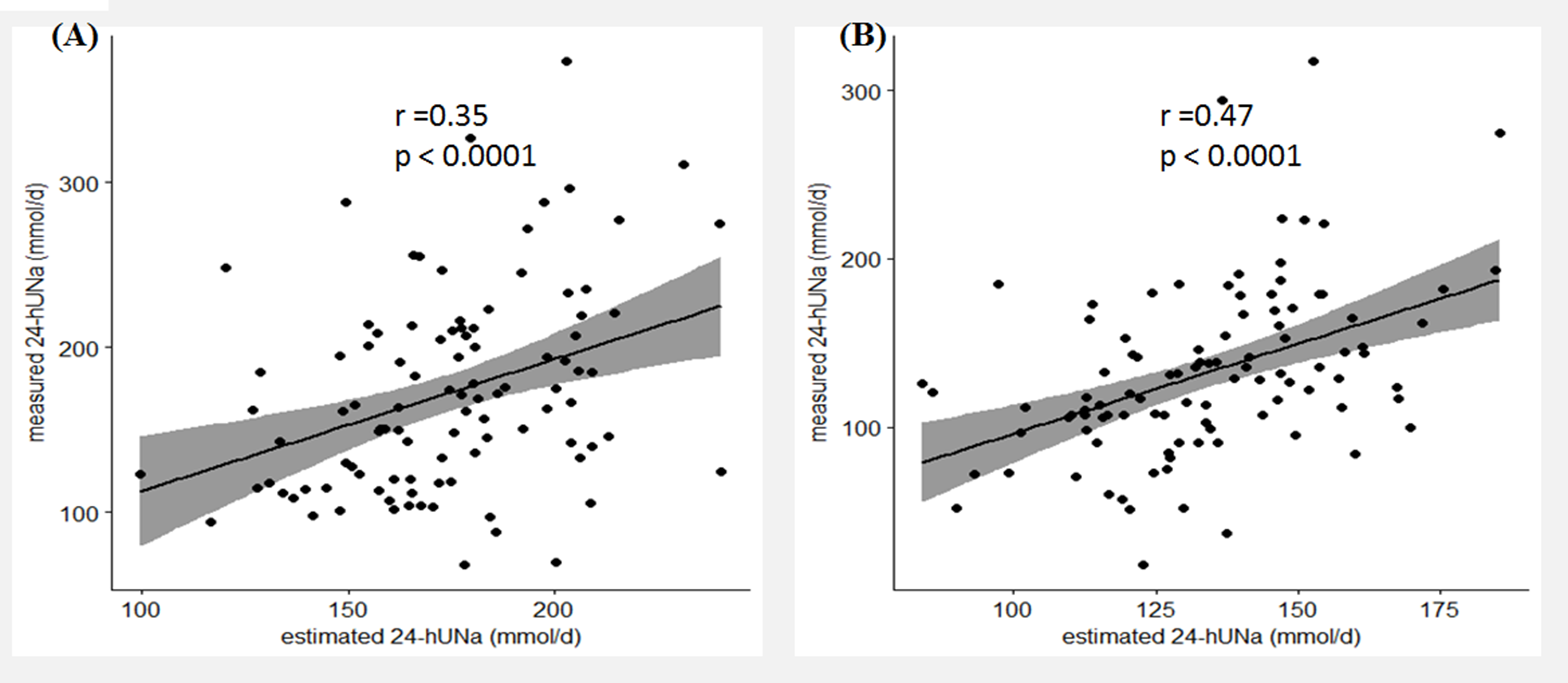

Supplement: S3 Fig — a Spearman’s correlation coefficient between measured 24-hUNa and other sodium-related variables b Measured 24-hUNa using 24-h urine (mmol/d) c Estimated 24-hUNa using equation (mmol/d) Panel (A): Estimated 24-hUNa (mmol/d), men; Panel (B): Estimated 24-hUNa (mmol/d), women. (TIF) [file pone.0192588.s003.tif]
