# Supplementary material for: Estimation model for habitual 24-hour urinary-sodium excretion using simple questionnaires from normotensive Koreans
Source: PLoS One. 2018 Feb 15;13(2):e0192588. doi: 10.1371/journal.pone.0192588 (PMC5813954; doi:10.1371/journal.pone.0192588)
Supplement: S1 Table — (DOCX) [file pone.0192588.s005.docx]

**S1 Table.** Summary of survey variables in training set (study 1) and validation set (study 2)

| Category | Study 1 | Study 2 | Remarks | Response, unit |
| --- | --- | --- | --- | --- |
| 1) Dietary measurements using questionnaire |  |  |  |  |
| Dietary behavior questionnaire | O | O | 20-items regarding behaviors of sodium and potassium intake | _ |
| 24-hour recall questionnaire | O, twice | × | Training set: average of the two measures, converted into the actual sodium intake amounts using CAN-pro Professional 4.0 | mg/day |
| SQ-FFQ | O | O | Investigated intake patterns, recently during 6 months |  |
| Salty-taste assessment | O | × | Evaluation using a salty-taste assessment kit |  |
| 2) Other measurements |  |  |  |  |
| a. Socio-demographic characteristics and several lifestyle factors |  |  |  |  |
| Age | O | O | _ | years |
| Regions | O | O | Residence | Seoul and Gyeonggi province (Central district), Chungcheong province (Middle district), Gyeongsang province (Southeast district), Jeolla province (Southwest district) |
| Smoking | O | O | Current smoking status | Non-smoker, past smoker, and current smoker |
| Regular exercise | O | O | Regular exercise was defined as more than 1 time per week and 30 min per one time. | Yes, No |
| Education | O | O | _ | ≤ High school graduate, ≥ College |
| Occupation | O | O | Final education |  |
| Household income | O | O | _ | ≤ 200, 200~300, 300~400, ≥ 400 (ten thousand won) |
| b. Anthropometric measurements |  |  |  |  |
| Body weight | O | O | Measured by the well-trained interviewers | Kilogram (kg) |
| Height | O | O | ” | Centimeter (cm) |
| Waist circumference | O | × | ” | Centimeter (cm) |
| Hip circumference | O | × | ” | Centimeter (cm) |
| Blood pressure | O | O | ”, SBP and DBP | mmHg |
| 3) Urine collection and analysis |  |  |  |  |
| 24-h urine sample collection | O, twice | O, one time | Training set: average of the two measures | mmol/d |
| 24-h urinary Sodium excretion | O, twice | O, one time | ” | mmol/d |
| 24-h urinary Potassium excretion | O, twice | O, one time | ” | g/d |
| 24-h urinary Creatinine excretion | O, twice | O, one time | ” | ml/d |
| 24-h urinary Volume | O, twice | O, one time | ” |  |
| Spot urine sample collection |  |  |  |  |
| Spot urinary Sodium excretion | O, twice | O, one time | Training set: average of the two measures | mmol/L |
