# Supplementary material for: Estimation model for habitual 24-hour urinary-sodium excretion using simple questionnaires from normotensive Koreans
Source: PLoS One. 2018 Feb 15;13(2):e0192588. doi: 10.1371/journal.pone.0192588 (PMC5813954; doi:10.1371/journal.pone.0192588)
Supplement: S2 Table — (DOCX) [file pone.0192588.s006.docx]

**S2 Table.** Univariate linear regression analysis for estimation of 24-hUNa (mmol/d), using β-coefficient estimates

| ***Men*** | | | | | | |  | |
| --- | --- | --- | --- | --- | --- | --- | --- | --- |
| Item | **Parameters** | | **β** | **SE** | **t-value** | ***P*-value** | | **VIF** |
|  | Age (year) | | 0.58 | 0.25 | 2.27 | 0.0236 | | 1.62 |
|  | Body weight (kg, logarithmic transformation) | | 156 | 25.8 | 6.04 | <.0001 | | 4.82 |
| M1_1 | Eating salty food? Not salty | | -22.8 | 9.66 | -2.36 | 0.0187 | | 1.22 |
| M1_2 | Eating salty food? Salty | | 13.5 | 7.77 | 1.74 | 0.0832 | | 1.28 |
| M2 | Kimchi consumption, < 1/day | | -40.4 | 9.95 | -4.06 | <.0001 | | 1.08 |
| M3 | Korean soup or stew consumption, ≥ 2/day | | 18.7 | 8.39 | 2.23 | 0.0267 | | 1.08 |
| M4 | Soy sauce or red pepper paste consumption, Occasionally | | -18.6 | 7.28 | -2.56 | 0.0111 | | 1.16 |
| M5 | Past smoker | | -4.69 | 8.28 | -0.57 | 0.5716 | | 1.15 |
|  | Waist circumference (cm, logarithmic transformation) | | 234 | 36.8 | 6.36 | <.0001 | | 3.93 |
|  | Regular exercise, yes | | -0.33 | 7.39 | -0.05 | 0.964 | | 1.13 |
|  | Salt preference, Very much like | | 9.34 | 7.58 | 1.23 | 0.2193 | | 1.78 |
|  | I eat the soup of noodles thoroughly., Slightly | | -18.5 | 8.87 | -2.08 | 0.0381 | | 3.25 |
|  | I eat the soup of noodles thoroughly., Moderately | | 1.61 | 8.12 | 0.20 | 0.8432 | | 3.87 |
|  | I eat the soup of noodles thoroughly., Very | | 11.1 | 8.07 | 1.37 | 0.1706 | | 4.00 |
|  | I eat the soup of noodles thoroughly., Extremely | | 27.1 | 10.9 | 2.49 | 0.0132 | | 2.85 |
|  | I ask to cook blandly when I eat out., Slightly | | -1.12 | 7.48 | -0.15 | 0.8816 | | 1.20 |
|  | I ask to cook blandly when I eat out., Moderately | | -7.09 | 15.5 | -0.46 | 0.6485 | | 1.21 |
|  | I ask to cook blandly when I eat out., Very | | 27.6 | 17.3 | 1.59 | 0.1119 | | 1.24 |
|  | I ask to cook blandly when I eat out., Extremely | | -18.0 | 46.6 | -0.39 | 0.7002 | | 1.09 |
|  | Eat out frequency for meal substitute, 1-2/week | | -11.0 | 7.52 | -1.46 | 0.1441 | | 2.49 |
|  | Eat out frequency for meal substitute, 3-4/week | | 1.67 | 9.54 | 0.18 | 0.8609 | | 2.13 |
|  | Eat out frequency for meal substitute, 4-5/week | | -1.20 | 11.6 | -0.10 | 0.9180 | | 1.69 |
|  | Eat out frequency for meal substitute, 1/day | | 2.22 | 11.0 | 0.20 | 0.8399 | | 1.74 |
|  | Eat out frequency for meal substitute, ≥ 2 /day | | 56.4 | 19.9 | 2.83 | 0.0049 | | 1.30 |
| ***Women*** | |  | | | | | | |
| Item | **Parameters** | | **β** | **SE** | **t-value** | ***P*-value** | | **VIF** |
|  | Age (year) | | 0.34 | 0.19 | 1.80 | 0.0735 | | 1.95 |
|  | Body weight (kg, logarithmic transformation) | | 110 | 20.2 | 5.42 | <.0001 | | 5.99 |
| F1 | Salt preference, Very much like | | 20.0 | 6.08 | 3.29 | 0.0011 | | 1.25 |
| F2 | Eating salty food? Salty | | 16.4 | 6.72 | 2.44 | 0.0153 | | 1.14 |
| F3 | Checking Na content for processed foods, No | | 8.35 | 6.40 | 1.31 | 0.1928 | | 1.07 |
| F4 | Nut consumption , Intermediate | | -15.3 | 5.53 | -2.76 | 0.0061 | | 1.07 |
| F5 | Past smoker | | -15.4 | 17.9 | -0.86 | 0.3878 | | 1.04 |
|  | Height (cm, logarithmic transformation) | | 24.2 | 77.4 | 0.31 | 0.7546 | | 2.21 |
|  | Waist circumference (cm, logarithmic transformation) | | 122 | 25.0 | 4.88 | <.0001 | | 3.46 |
|  | Regular exercise, yes | | 2.13 | 5.58 | 0.38 | 0.7024 | | 1.13 |
|  | I eat the soup of noodles thoroughly., Slightly | | -0.57 | 6.13 | -0.09 | 0.9259 | | 2.30 |
|  | I eat the soup of noodles thoroughly., Moderately | | 4.31 | 6.10 | 0.71 | 0.4804 | | 2.49 |
|  | I eat the soup of noodles thoroughly., Very | | 12.6 | 7.31 | 1.72 | 0.0866 | | 2.23 |
|  | I eat the soup of noodles thoroughly., Extremely | | 29.4 | 12.3 | 2.39 | 0.0176 | | 1.69 |
|  | I eat often (e.g. ramen, fish cake, canned food)., Slightly | | 0.37 | 5.62 | 0.07 | 0.9478 | | 2.80 |
|  | I eat often (e.g. ramen, fish cake, canned food)., Moderately | | -0.74 | 6.07 | -0.12 | 0.9027 | | 3.22 |
|  | I eat often (e.g. ramen, fish cake, canned food)., Very | | -3.73 | 9.72 | -0.38 | 0.7014 | | 2.33 |
|  | I eat often (e.g. ramen, fish cake, canned food)., Extremely | | 9.00 | 25.1 | 0.36 | 0.7204 | | 1.76 |
|  | I often eat fish or fish with salt., Slightly | | -1.13 | 7.88 | -0.14 | 0.8858 | | 6.32 |
|  | I often eat fish or fish with salt., Moderately | | -9.67 | 5.58 | -1.73 | 0.0838 | | 11.17 |
|  | I often eat fish or fish with salt., Very | | 10.6 | 6.27 | 1.68 | 0.0932 | | 9.29 |
|  | I often eat fish or fish with salt., Extremely | | 10.8 | 9.28 | 1.17 | 0.2436 | | 5.10 |
|  | I eat instant food often (e.g. hamburger, pizza, etc...)., Slightly | | 0.49 | 5.58 | 0.09 | 0.9307 | | 3.41 |
|  | I eat instant food often (e.g. hamburger, pizza, etc...)., Moderately | | 1.50 | 5.96 | 0.25 | 0.8020 | | 3.67 |
|  | I eat instant food often (e.g. hamburger, pizza, etc...)., Very | | 3.61 | 11.0 | 0.33 | 0.7436 | | 1.88 |
|  | I eat instant food often (e.g. hamburger, pizza, etc...)., Extremely | | 10.2 | 29.0 | 0.35 | 0.7262 | | 1.36 |
|  | I eat potato chips, crackers and cookies often as a snack., Slightly | | -8.61 | 5.83 | -1.48 | 0.1407 | | 6.15 |
|  | I eat potato chips, crackers and cookies often as a snack., Moderately | | -2.10 | 5.79 | -0.36 | 0.7169 | | 6.43 |
|  | I eat potato chips, crackers and cookies often as a snack., Very | | 9.89 | 7.00 | 1.41 | 0.1586 | | 4.88 |
|  | I eat potato chips, crackers and cookies often as a snack., Extremely | | 9.01 | 14.7 | 0.61 | 0.5396 | | 2.06 |
|  | I use low salt-product (low-salt soy paste, low-salt soy sauce), Slightly | | 5.24 | 5.68 | 0.92 | 0.3575 | | 1.92 |
|  | I use low salt-product (low-salt soy paste, low-salt soy sauce), Moderately | | -5.32 | 7.50 | -0.71 | 0.4789 | | 1.81 |
|  | I use low salt-product (low-salt soy paste, low-salt soy sauce), Very | | -5.79 | 9.43 | -0.61 | 0.5396 | | 1.67 |
|  | I use low salt-product (low-salt soy paste, low-salt soy sauce), Extremely | | -18.0 | 17.8 | -1.01 | 0.3138 | | 1.36 |
|  | I ask to cook blandly when I eat out., Slightly | | 7.91 | 6.04 | 1.31 | 0.1912 | | 1.42 |
|  | I ask to cook blandly when I eat out., Moderately | | -6.68 | 9.87 | -0.68 | 0.4988 | | 1.20 |
|  | I ask to cook blandly when I eat out., Very | | 10.8 | 12.8 | 0.85 | 0.3976 | | 1.40 |
|  | I ask to cook blandly when I eat out., Extremely | | -18.0 | 25.1 | -0.72 | 0.4746 | | 1.88 |
|  | I often eat stewed food or stir-fried food as a side dish., Slightly | | -9.5 | 6.10 | -1.56 | 0.1199 | | 2.14 |
|  | I often eat stewed food or stir-fried food as a side dish., Moderately | | -3.80 | 6.05 | -0.63 | 0.5304 | | 2.15 |
|  | I often eat stewed food or stir-fried food as a side dish., Very | | 22.6 | 8.17 | 2.77 | 0.0060 | | 2.02 |
|  | I often eat stewed food or stir-fried food as a side dish., Extremely | | -3.18 | 15.3 | -0.21 | 0.8357 | | 2.21 |

β, beta coefficients; SE, standard error; VIF, variance inflation factor; 24-hUNa, 24-h urinary-sodium excretion
