# Supplementary material for: Estimation model for habitual 24-hour urinary-sodium excretion using simple questionnaires from normotensive Koreans
Source: PLoS One. 2018 Feb 15;13(2):e0192588. doi: 10.1371/journal.pone.0192588 (PMC5813954; doi:10.1371/journal.pone.0192588)
Supplement: S3 Table — (DOCX) [file pone.0192588.s007.docx]

**S3 Table.** Gender-specific regression equations estimating 24-hUNa from age, weight, smoking status, and simple dietary questionnaire items

| **For Men** | | |  |
| --- | --- | --- | --- |
| Eating salty food | “Do you think you eat salty food?” | 1. Usually bland | Not salty |
|  |  | 2. Usually a little bland |  |
|  |  | 3. Regular salt (especially prefer not eating salty or bland food) |  |
|  |  | 4. A little salty | Salty |
|  |  | 5. Very salty |  |
| Kimchi consumption | “How often do you eat Kimchi?” | 1. ≥3/d |  |
|  |  | 2. 2/d |  |
|  |  | 3. 1/d |  |
|  |  | 4. 3-6/week | < 1/day |
|  |  | 5. 1-2/week |  |
|  |  | 6. 1-3/month |  |
|  |  | 7. 1/month or never |  |
| Korean soup or stew consumption | “How often do you eat Korean soup or stew?” | 1. ≥3/d | ≥2/day |
|  |  | 2. 2/d |  |
|  |  | 3. 1/d |  |
|  |  | 4. 3-6/week |  |
|  |  | 5. 1-2/week |  |
|  |  | 6. 1-3/month |  |
|  |  | 7. 1/month or never |  |
| Soy sauce or red pepper paste consumption | “Do you add a lot of soy sauce or red pepper paste to sliced raw fish, jeon (Korean pancake), or fried food?” | 1. Not at all | Occasionally |
|  |  | 2. Do not often do that |  |
|  |  | 3. Neutral |  |
|  |  | 4. Like |  |
|  |  | 5. Strongly like |  |
| **For women** | | |  |
| Salt preference | “Do you like salty food?” | 1. Strongly dislike |  |
|  |  | 2. Dislike |  |
|  |  | 3. Neutral |  |
|  |  | 4. Like | Very much like |
|  |  | 5. Strongly like |  |
| Eating salty food | “Do you think you eat salty food?” | 1. Usually bland |  |
|  |  | 2. Usually a little bland |  |
|  |  | 3. Regular salt (especially prefer not eating salty or bland food) | Salty |
|  |  | 4. A little salty |  |
|  |  | 5. Very salty |  |
| Checking Na content for processed foods | “Do you check the sodium content when you buy processed foods?” | 1. Not at all | No |
|  |  | 2. Do not often do that |  |
|  |  | 3. Normal (sometimes confirm & sometimes exercise caution) |  |
|  |  | 4. Usually confirm |  |
|  |  | 5. Always confirm |  |
| Nut consumption | “How often do you eat nuts for a snack?” | 1. ≥3/d |  |
|  |  | 2. 2/d |  |
|  |  | 3. 1/d | Intermediate |
|  |  | 4. 3-6/week |  |
|  |  | 5. 1-2/week |  |
|  |  | 6. 1-3/month |  |
|  |  | 7. 1/month or never |  |
