# Supplementary material for: Estimation model for habitual 24-hour urinary-sodium excretion using simple questionnaires from normotensive Koreans
Source: PLoS One. 2018 Feb 15;13(2):e0192588. doi: 10.1371/journal.pone.0192588 (PMC5813954; doi:10.1371/journal.pone.0192588)
Supplement: S1 Survey Questionnaire — (DOCX) [file pone.0192588.s008.docx]

**설문지 양식**

| 1. 일반사항 조사지  2. 신체계측 조사지  3. 식행동 조사지  4. 식품섭취빈도 조사지  5. 24시간 회상법 조사지 |
| --- |

| **조사 지역** | **조사담당자** | **조사대상자** | **조사일** |
| --- | --- | --- | --- |
|  |  |  |  |

안녕하십니까? 바쁘신 와중에도 귀중한 시간을 내주셔서 감사드립니다.

본 설문의 내용은 연구 자료로만 활용될 것이며, 일체의 사항이나 응답한 내용들로 인한 어떠한 불이익도 없음을 말씀드립니다. 본 조사는 통계법 제13조 및 제14조에 의거 비밀이 보장되며 통계자료 목적 이외에는 사용하지 않습니다.

**일반사항 조사지**

**※ 아래 문항은 귀하의 일상생활 및 신체활동에 관한 것입니다.**

| **1. 귀하의 일상생활 활동은 다음 중 어느 것에 가깝습니까?**  ① 안정 상태 (거의 누워 있거나 앉아서 지냄)  ② 가벼운 활동 (사무관리 ‧ 기술직종사자, 가사 노동시간이 적은 주부, 이와 유사한 직종)  ③ 보통 활동 (가사작업량이 많은 주부, 제조업 ‧ 가공업 ‧ 판매업종사자, 교사 등)  ④ 심한 활동 (농업 ‧ 어업 ‧ 토목업 ‧ 건축업종사자, 이와 유사한 내용의 직종)  ⑤ 격심한 활동 (운동선수, 목재운반, 농번기 농업종사자등과 유사한 힘쓰는 육체 노동직종)  **2. 최근 일주일 동안 평소보다 몸이 매우 힘들거나 숨이 많이 가쁜 격렬한 신체활동을**  **10분 이상한 날은 며칠입니까?**  ① 전혀 하지 않음(☞3번으로) ②주 1~2회 ③주 3~4회 ④주 5~6회 ⑤ 매일  **2-1. 이러한 격렬한 신체활동을 한 날, 보통 하루에 몇 분간 했습니까?**  하루에 () 시간 () 분  **3. 최근 일주일 동안 평소보다 숨이 약간 가쁜 중등도 신체활동을 10분 이상 한 날은 며칠입니까?** ① 전혀 하지 않음(☞4번으로) ②주 1~2회 ③주 3~4회 ④주 5~6회 ⑤ 매일  **3-1. 이러한 중등도 신체활동을 한 날, 보통 하루에 몇 분간 했습니까?**  하루에 () 시간 () 분  **4. 최근 일주일 동안 한 번에 적어도 10분 이상 걸은 날은 며칠입니까?**  ① 전혀 하지 않음 ②주 1~2회 ③주 3~4회 ④주 5~6회 ⑤ 매일  **4-1. 이러한 날 중 하루 동안 걷는 시간은 보통 얼마나 됩니까?**  하루에 () 시간 () 분  **5. 현재 담배를 피우십니까?**  ① 매일 피움 ( ) 개피/일 ② 가끔 피움 ( )개피/일  ③ 과거에는 피웠으나 현재 피우지 않음(흡연기간: ) ④ 피운 적 없음 |
| --- |

**신체계측 조사지(조사자용)**

| **번호** | **이름** | **신장 (cm)** | **체중 (kg)** | **허리둘레 (cm)** | **엉덩이둘레 (cm)** | **혈압 (mm/Hg)** |
| --- | --- | --- | --- | --- | --- | --- |
|  |  |  |  |  |  |  |
|  |  |  |  |  |  |  |
|  |  |  |  |  |  |  |
|  |  |  |  |  |  |  |
|  |  |  |  |  |  |  |
|  |  |  |  |  |  |  |
|  |  |  |  |  |  |  |
|  |  |  |  |  |  |  |
|  |  |  |  |  |  |  |
|  |  |  |  |  |  |  |
|  |  |  |  |  |  |  |
|  |  |  |  |  |  |  |
|  |  |  |  |  |  |  |
|  |  |  |  |  |  |  |
|  |  |  |  |  |  |  |
|  |  |  |  |  |  |  |
|  |  |  |  |  |  |  |
|  |  |  |  |  |  |  |
|  |  |  |  |  |  |  |
|  |  |  |  |  |  |  |
|  |  |  |  |  |  |  |
|  |  |  |  |  |  |  |
|  |  |  |  |  |  |  |
|  |  |  |  |  |  |  |
|  |  |  |  |  |  |  |
|  |  |  |  |  |  |  |

**식행동 조사지**

**※ 아래 식행동 문항을 읽고 해당하는 곳에 체크해 주십시오.**

| **식행동 문항** | | | 전혀  그렇지  않다 | | | 그렇지  않다 | | 보통  이다 | | | | 그렇다 | | 매우  그렇다 | |
| --- | --- | --- | --- | --- | --- | --- | --- | --- | --- | --- | --- | --- | --- | --- | --- |
| 1. 국이나 국수류의 국물을 남김없이 먹는다. | | |  | | |  | |  | | | |  | |  | |
| 2. 라면, 어묵, 통조림류 등 가공식품을 자주 먹는다. | | |  | | |  | |  | | | |  | |  | |
| 3. 자주(주 2~3회) 외식하거나 배달시켜 먹는다. | | |  | | |  | |  | | | |  | |  | |
| 4. 생선자반이나 소금을 뿌린 생선을 자주 먹는다. | | |  | | |  | |  | | | |  | |  | |
| 5. 햄버거, 피자 등 인스턴트식품을 자주 먹는다. | | |  | | |  | |  | | | |  | |  | |
| 6. 간식으로 포테이토칩, 스낵, 크래커 등을 자주 먹는다. | | |  | | |  | |  | | | |  | |  | |
| 7. 식사 전에 습관적으로 음식에 소금이나 간장을 넣는다. | | |  | | |  | |  | | | |  | |  | |
| 8. 저염간장, 저염된장 등 저염제품을 사용한다. | | |  | | |  | |  | | | |  | |  | |
| 9. 외식할 때 싱겁게 해달라고 요구한다. | | |  | | |  | |  | | | |  | |  | |
| 10. 반찬으로는 조림류나 볶음류를 자주 먹는다. | | |  | | |  | |  | | | |  | |  | |
| 11. 양념된 고기나 해물, 판매하는 밑반찬류를 자주 구입한다. | | |  | | |  | |  | | | |  | |  | |
| 12. 쌀밥과 잡곡 위주의 한식을 먹는다. | | |  | | |  | |  | | | |  | |  | |
| 13. 채소류 반찬을 매끼 많이 먹는다. | | |  | | |  | |  | | | |  | |  | |
| 14. 감자나 콩, 두부로 만든 음식을 자주 먹는다. | | |  | | |  | |  | | | |  | |  | |
| 15. 과일을 매일 먹는다. | | |  | | |  | |  | | | |  | |  | |
|  | | | 전혀 그렇지 않다 | | | 그렇지 않다 | | 보통이다 | | | | 그렇다 | | 항상 그렇다 | |
| 16. 고추장, 간장을 듬뿍 찍어 먹는다(회, 전, 튀김 등에). | | |  | | |  | |  | | | |  | |  | |
| 17. 가공식품 구입 시 나트륨 함량을 확인한다. | | |  | | |  | |  | | | |  | |  | |
| **문항 (최근 1년 동안 식품섭취빈도)** | | | 하루 | | | | | | | 1주 | | | 1달 | | |
|  |  |  | 3 | | 2 | | 1 | | | 3~6 | 1~2 | | 1~3 | | 1회미만 |
| 18. 김치류를 얼마나 자주 드십니까? | | |  | |  | |  | | |  |  | |  | |  |
| 19. 국, 찌개를 얼마나 자주 드십니까? | | |  | |  | |  | | |  |  | |  | |  |
| 20. 견과류(땅콩 등)를 간식으로 얼마나 자주 드십니까? | | |  | |  | |  | | |  |  | |  | |  |
| **식사대용**  **외식 빈도**  **(배달 포함)** | ① 거의 하지 않는다  ④ 주 5-6회 | ② 주 1-2회  ⑤ 매일 1회 | | | | | | | ③ 주 3-4회  ⑥ 매일 2회 이상 | | | | | | |
| **외식 시**  **(배달 포함)**  **자주 먹는**  **음식**  **(3개 선택)** | ① 직원식당 (단체급식)  ③ 탕, 찌개류  ⑤ 가정식 백반류 (밥, 반찬 형태)  ⑦ 볶음밥, 덮밥, 조림류  (오징어볶음, 생선조림 등) | | | ② 짜장면, 짬뽕 등의 중국음식  ④ 떡볶이, 라면 등의 분식류  ⑥ 샌드위치, 토스트  ⑧ 햄버거, 피자, 파스타 등  ⑨ 고기집(삼겹살,로스구이, 갈비 등) | | | | | | | | | | | |
| **외식 선택 시**  **고려사항**  **(1개 선택)** | ① 맛  ④ 저염식 여부 | ② 건강식 여부  ⑤ 위생 | | | | | | | ③ 가격  ⑥ 식사 준비시간 (배달 시간) | | | | | | |

**식품섭취빈도 조사지**

**※ 다음 식품 혹은 각 식품을 주재료로 조리한 음식을 최근 6개월 동안 얼마나 자주 드셨는지 응답해 주십시오.**

| **음식의 종류** | | | **보통 기준량** | **실제섭취량** | | | **섭취빈도** | | | | | | | | |
| --- | --- | --- | --- | --- | --- | --- | --- | --- | --- | --- | --- | --- | --- | --- | --- |
|  |  |  |  |  |  |  | **하루** | | | **1주** | | | **1달** | | **안먹음** |
|  |  |  |  | **이상** | **보통** | **이하** | **3회** | **2회** | **1회** | **5-6회** | **3-4회** | **1-2회** | **2-3회** | **1회** |  |
| **밥류** | **1** | **김치볶음밥** | **1접시(160g)** |  |  |  |  |  |  |  |  |  |  |  |  |
|  | **2** | **김밥** | **1줄(140g)** |  |  |  |  |  |  |  |  |  |  |  |  |
|  | **3** | **비빔밥** | **1그릇(240g)** |  |  |  |  |  |  |  |  |  |  |  |  |
|  | **4** | **카레라이스** | **1그릇(230g)** |  |  |  |  |  |  |  |  |  |  |  |  |
|  | **5** | **덮밥종류** | **1그릇(200g)** |  |  |  |  |  |  |  |  |  |  |  |  |
|  | **6** | **죽종류** | **1그릇(80g)** |  |  |  |  |  |  |  |  |  |  |  |  |
| **국수류** | **7** | **라면류** | **1그릇(140g)** |  |  |  |  |  |  |  |  |  |  |  |  |
|  | **8** | **칼국수** | **1그릇(170g)** |  |  |  |  |  |  |  |  |  |  |  |  |
|  | **9** | **칼국수**  **+ 양념장** | **1그릇(170g) +양념장(15g)** |  |  |  |  |  |  |  |  |  |  |  |  |
|  | **10** | **비빔국수** | **1그릇(170g)** |  |  |  |  |  |  |  |  |  |  |  |  |
|  | **11,** | **잔치국수** | **1그릇(190g)** |  |  |  |  |  |  |  |  |  |  |  |  |
|  | **12** | **잔치국수**  **+ 양념장** | **1그릇(170g) +양념장(15g)** |  |  |  |  |  |  |  |  |  |  |  |  |
|  | **13** | **메밀국수** | **1그릇(190g)** |  |  |  |  |  |  |  |  |  |  |  |  |
|  | **14** | **물냉면** | **1그릇(160g)** |  |  |  |  |  |  |  |  |  |  |  |  |
|  | **15** | **비빔냉면** | **1그릇(200g)** |  |  |  |  |  |  |  |  |  |  |  |  |
|  | **16** | **우동 혹은 짬뽕** | **1그릇(150g)** |  |  |  |  |  |  |  |  |  |  |  |  |
|  | **17** | **자장면** | **1그릇(110g)** |  |  |  |  |  |  |  |  |  |  |  |  |
| **빵류** | **18** | **샌드위치** | **1개(130g)** |  |  |  |  |  |  |  |  |  |  |  |  |
|  | **19** | **피자** | **1조각(150g)** |  |  |  |  |  |  |  |  |  |  |  |  |
| **국,**  **찌개, 탕** | **20** | **된장국** | **1대접(100g)** |  |  |  |  |  |  |  |  |  |  |  |  |
|  | **21** | **미역국** | **1대접(32g)** |  |  |  |  |  |  |  |  |  |  |  |  |
|  | **22** | **김치국** | **1대접(40g)** |  |  |  |  |  |  |  |  |  |  |  |  |
|  | **23** | **콩나물국** | **1대접(40g)** |  |  |  |  |  |  |  |  |  |  |  |  |
|  | **24** | **쇠고기국** | **1대접(70g)** |  |  |  |  |  |  |  |  |  |  |  |  |
|  | **25** | **된장찌개** | **1대접(100g)** |  |  |  |  |  |  |  |  |  |  |  |  |
|  | **26** | **김치찌개** | **1대접(120g)** |  |  |  |  |  |  |  |  |  |  |  |  |
|  | **27** | **생선찌개** | **1대접(130g)** |  |  |  |  |  |  |  |  |  |  |  |  |
|  | **28** | **순두부찌개** | **1대접(180g)** |  |  |  |  |  |  |  |  |  |  |  |  |

| **음식의 종류** | | | **보통 기준량** | **실제섭취량** | | | **섭취빈도** | | | | | | | | |
| --- | --- | --- | --- | --- | --- | --- | --- | --- | --- | --- | --- | --- | --- | --- | --- |
|  |  |  |  |  |  |  | **하루** | | | **1주** | | | **1달** | | **안먹음** |
|  |  |  |  | **이상** | **보통** | **이하** | **3회** | **2회** | **1회** | **5-6회** | **3-4회** | **1-2회** | **2-3회** | **1회** |  |
| **국,**  **찌개, 탕** | **29** | **생선통조림찌개** | **1대접(120g)** |  |  |  |  |  |  |  |  |  |  |  |  |
|  | **30** | **오징어찌개** | **1대접(140g)** |  |  |  |  |  |  |  |  |  |  |  |  |
|  | **31** | **감자탕** | **1대접(110g)** |  |  |  |  |  |  |  |  |  |  |  |  |
|  | **32** | **곰탕(사골탕)** | **1대접(90g)** |  |  |  |  |  |  |  |  |  |  |  |  |
|  | **33** | **육개장** | **1대접(160g)** |  |  |  |  |  |  |  |  |  |  |  |  |
|  | **34** | **해물탕** | **1대접(110g)** |  |  |  |  |  |  |  |  |  |  |  |  |
|  | **35** | **어묵탕** | **1대접(130g)** |  |  |  |  |  |  |  |  |  |  |  |  |
| **어패류**  **반찬** | **36** | **생선구이** | **1토막(60g)** |  |  |  |  |  |  |  |  |  |  |  |  |
|  | **37** | **멸치볶음** | **1종지(30g)** |  |  |  |  |  |  |  |  |  |  |  |  |
|  | **38** | **어묵볶음** | **1접시(15.5g)** |  |  |  |  |  |  |  |  |  |  |  |  |
|  | **39** | **오징어채**  **볶음** | **1접시(30g)** |  |  |  |  |  |  |  |  |  |  |  |  |
|  | **40** | **자반고등어조림** | **1접시(110g)** |  |  |  |  |  |  |  |  |  |  |  |  |
|  | **41** | **생선튀김** | **1토막(70g)** |  |  |  |  |  |  |  |  |  |  |  |  |
|  | **42** | **생선전** | **1쪽(100g)** |  |  |  |  |  |  |  |  |  |  |  |  |
|  | **43** | **생선전**  **+ 초간장** | **1쪽(100g)**  **+초간장(15g)** |  |  |  |  |  |  |  |  |  |  |  |  |
|  | **44** | **해물전** | **1장(110g)** |  |  |  |  |  |  |  |  |  |  |  |  |
|  | **45** | **오징어낙지볶음** | **1접시(120g)** |  |  |  |  |  |  |  |  |  |  |  |  |
| **고기,**  **알,**  **콩류**  **반찬** | **46** | **달걀찜** | **1개(50g)** |  |  |  |  |  |  |  |  |  |  |  |  |
|  | **47** | **돼지고기**  **볶음** | **1접시(100g)** |  |  |  |  |  |  |  |  |  |  |  |  |
|  | **48** | **편육(보쌈)** | **1접시(120g)** |  |  |  |  |  |  |  |  |  |  |  |  |
|  | **49** | **두부조림** | **3쪽(60g)** |  |  |  |  |  |  |  |  |  |  |  |  |
|  | **50** | **순대** | **1접시(90g)** |  |  |  |  |  |  |  |  |  |  |  |  |
|  | **51** | **순대+소금** | **1접시(90g) + 소금(1g)** |  |  |  |  |  |  |  |  |  |  |  |  |
|  | **52** | **순대+된장** | **1접시(90g)**  **+ 된장(20g)** |  |  |  |  |  |  |  |  |  |  |  |  |
|  | **53** | **돈가스** | **2쪽(100g)** |  |  |  |  |  |  |  |  |  |  |  |  |
|  | **54** | **돈가스**  **+ 소스** | **2쪽(100g)**  **+ 소스(20g)** |  |  |  |  |  |  |  |  |  |  |  |  |
|  | **55** | **닭찜** | **1접시(120g)** |  |  |  |  |  |  |  |  |  |  |  |  |
|  | **56** | **닭볶음탕** | **1접시(120g)** |  |  |  |  |  |  |  |  |  |  |  |  |
|  | **57** | **돼지갈비찜** | **4쪽(100g)** |  |  |  |  |  |  |  |  |  |  |  |  |
|  | **58** | **삼겹살**  **+ 소금** | **3장(50g)**  **+ 소금(14g)** |  |  |  |  |  |  |  |  |  |  |  |  |
|  | **59** | **삼겹살**  **+ 쌈장** | **3장(50g)**  **+ 쌈장(20g)** |  |  |  |  |  |  |  |  |  |  |  |  |

| **음식의 종류** | | | **보통 기준량** | **실제섭취량** | | | **섭취빈도** | | | | | | | | |
| --- | --- | --- | --- | --- | --- | --- | --- | --- | --- | --- | --- | --- | --- | --- | --- |
|  |  |  |  |  |  |  | **하루** | | | **1주** | | | **1달** | | **안먹음** |
|  |  |  |  | **이상** | **보통** | **이하** | **3회** | **2회** | **1회** | **5-6회** | **3-4회** | **1-2회** | **2-3회** | **1회** |  |
| **고기,**  **알,**  **콩류 반찬** | **60** | **불고기** | **1접시(100g)** |  |  |  |  |  |  |  |  |  |  |  |  |
|  | **61** | **닭튀김** | **2쪽(70g)** |  |  |  |  |  |  |  |  |  |  |  |  |
| **나물, 생채류** | **62** | **김구이** | **8장(4g)** |  |  |  |  |  |  |  |  |  |  |  |  |
|  | **63** | **쌈 + 쌈장** | **쌈장(25g)** |  |  |  |  |  |  |  |  |  |  |  |  |
|  | **64** | **시금치나물** | **1접시(70g)** |  |  |  |  |  |  |  |  |  |  |  |  |
|  | **65** | **채소부침개 혹은 전** | **1장(70g)** |  |  |  |  |  |  |  |  |  |  |  |  |
|  | **66** | **채소부침개,전 + 간장** | **1장(70g)**  **+ 간장(15g)** |  |  |  |  |  |  |  |  |  |  |  |  |
| **김치류** | **67** | **배추김치** | **1보시기(40g)** |  |  |  |  |  |  |  |  |  |  |  |  |
|  | **68** | **총각김치** | **1보시기(50g)** |  |  |  |  |  |  |  |  |  |  |  |  |
|  | **69** | **열무김치** | **1보시기(40g)** |  |  |  |  |  |  |  |  |  |  |  |  |
|  | **70** | **물김치류** | **1대접(80g)** |  |  |  |  |  |  |  |  |  |  |  |  |
|  | **71** | **김치볶음** | **1접시(160g)** |  |  |  |  |  |  |  |  |  |  |  |  |
|  | **72** | **김치전** | **1장(100g)** |  |  |  |  |  |  |  |  |  |  |  |  |
| **장아찌**  **또는**  **젓갈류** | **73** | **오이소박이** | **2개(70g)** |  |  |  |  |  |  |  |  |  |  |  |  |
|  | **74** | **마늘장아찌** | **1종지(20g)** |  |  |  |  |  |  |  |  |  |  |  |  |
|  | **75** | **양파장아찌** | **1종지(20g)** |  |  |  |  |  |  |  |  |  |  |  |  |
|  | **76** | **오징어젓** | **1종지(20g)** |  |  |  |  |  |  |  |  |  |  |  |  |
| **양념류** | **77** | **(튀김류) + 토마토케첩** | **1Ts(16g)** |  |  |  |  |  |  |  |  |  |  |  |  |
|  | **78** | **식탁에서 치는 소금** | **1Ts(8g)** |  |  |  |  |  |  |  |  |  |  |  |  |

**• 연령: 세 · • 성별:** □ 남 □ 여

**짠 음식을 좋아하십니까?** □ 예 □ 아니오

**평상시 짜게 드신다고 생각하십니까?**

① 싱겁게 먹는 편

② 약간 싱겁게 먹는 편

③ 보통으로 먹는 편

④ 약간 짜게 먹는 편

⑤ 짜게 먹는 편

| **학 력** | ① 초등학교 졸업  ④ 대학교 졸업 | ② 중학교 졸업  ⑤ 대학원 이상 | ③ 고등학교 졸업 |
| --- | --- | --- | --- |
| **직 업** | ① 전문직  ④ 주부 | ② 유통업/서비스업/자영업  ⑤ 회사원 | ③ 공무원  ⑥ 대학생 / 기타 |
| **월 평균**  **수 입** | ① 100만원 미만  ④ 300~400만원 미만 | ② 100~200만원 미만  ⑤ 400~500만원 미만 | ③ 200~300만원 미만  ⑥ 500만원 이상 |

**24시간 회상법 조사지(1일차)**

**어제 하루 동안 섭취하신 식사 내용과 분량을 말씀해 주십시오(식이보충제 등도 포함).**

| **식사구분** | **시간** | **식사장소** | **음식명** | **섭취량** | | | **재료명** | **재료량** | | | **비고** | |
| --- | --- | --- | --- | --- | --- | --- | --- | --- | --- | --- | --- | --- |
|  |  |  |  | **눈대**  **중량** | **부피** | **중량** |  | **눈대**  **중량** | **부피** | **중량** | **제품명** | **제조 회사명** |
|  |  |  |  |  |  |  |  |  |  |  |  |  |
|  |  |  |  |  |  |  |  |  |  |  |  |  |
|  |  |  |  |  |  |  |  |  |  |  |  |  |
|  |  |  |  |  |  |  |  |  |  |  |  |  |
|  |  |  |  |  |  |  |  |  |  |  |  |  |
|  |  |  |  |  |  |  |  |  |  |  |  |  |
|  |  |  |  |  |  |  |  |  |  |  |  |  |
|  |  |  |  |  |  |  |  |  |  |  |  |  |
|  |  |  |  |  |  |  |  |  |  |  |  |  |
|  |  |  |  |  |  |  |  |  |  |  |  |  |
|  |  |  |  |  |  |  |  |  |  |  |  |  |
|  |  |  |  |  |  |  |  |  |  |  |  |  |
|  |  |  |  |  |  |  |  |  |  |  |  |  |
|  |  |  |  |  |  |  |  |  |  |  |  |  |
|  |  |  |  |  |  |  |  |  |  |  |  |  |
|  |  |  |  |  |  |  |  |  |  |  |  |  |
|  |  |  |  |  |  |  |  |  |  |  |  |  |
|  |  |  |  |  |  |  |  |  |  |  |  |  |
|  |  |  |  |  |  |  |  |  |  |  |  |  |
|  |  |  |  |  |  |  |  |  |  |  |  |  |

2. 어제 섭취하신 식사 분량은 평소의 식사에 비해서 어떻습니까?

① 평소에 비해서 많이 섭취하였다 ② 평소와 비슷하였다 ③ 평소에 비해 적게 섭취하였다3. 하루에 물[생수, 보리차, 결명자차, 옥수수차 등]을 얼마나 섭취하십니까? ( )컵(200ml)

**24시간 회상법 조사지(5일차)**

**어제 하루 동안 섭취하신 식사 내용과 분량을 말씀해 주십시오(식이보충제 등도 포함).**

| **식사구분** | **시간** | **식사장소** | **음식명** | **섭취량** | | | **재료명** | **재료량** | | | **비고** | |
| --- | --- | --- | --- | --- | --- | --- | --- | --- | --- | --- | --- | --- |
|  |  |  |  | **눈대**  **중량** | **부피** | **중량** |  | **눈대**  **중량** | **부피** | **중량** | **제품명** | **제조 회사명** |
|  |  |  |  |  |  |  |  |  |  |  |  |  |
|  |  |  |  |  |  |  |  |  |  |  |  |  |
|  |  |  |  |  |  |  |  |  |  |  |  |  |
|  |  |  |  |  |  |  |  |  |  |  |  |  |
|  |  |  |  |  |  |  |  |  |  |  |  |  |
|  |  |  |  |  |  |  |  |  |  |  |  |  |
|  |  |  |  |  |  |  |  |  |  |  |  |  |
|  |  |  |  |  |  |  |  |  |  |  |  |  |
|  |  |  |  |  |  |  |  |  |  |  |  |  |
|  |  |  |  |  |  |  |  |  |  |  |  |  |
|  |  |  |  |  |  |  |  |  |  |  |  |  |
|  |  |  |  |  |  |  |  |  |  |  |  |  |
|  |  |  |  |  |  |  |  |  |  |  |  |  |
|  |  |  |  |  |  |  |  |  |  |  |  |  |
|  |  |  |  |  |  |  |  |  |  |  |  |  |
|  |  |  |  |  |  |  |  |  |  |  |  |  |
|  |  |  |  |  |  |  |  |  |  |  |  |  |
|  |  |  |  |  |  |  |  |  |  |  |  |  |
|  |  |  |  |  |  |  |  |  |  |  |  |  |
|  |  |  |  |  |  |  |  |  |  |  |  |  |

2. 어제 섭취하신 식사 분량은 평소의 식사에 비해서 어떻습니까?

① 평소에 비해서 많이 섭취하였다 ② 평소와 비슷하였다 ③ 평소에 비해 적게 섭취하였다3. 하루에 물[생수, 보리차, 결명자차, 옥수수차 등]을 얼마나 섭취하십니까? ( )컵(200ml)

**Questionaries form (English)**

| 1. General characteristics questionnaire  2. Anthropometric measurements questionnaire  3. Dietary behavior questionnaire  4. Food frequency questionaries  5. 24-hour recall questionaries |
| --- |

| **Survey Location** | **Survey Director** | **Survey Subject** | **Survey date** |
| --- | --- | --- | --- |
|  |  |  |  |

Hi, thank you for give time in your busy day. This survey will use for only research data and there are no disadvantage based on your answer and contents of survey. This survey is not use any other purpose except statistics data according to statistic law article 13 and article 14

**General Survey**

**※ This survey is about daily life and body activities**

| **1. Which is most close to your daily life activities?**  ① Stable (Mostly stay down or sit down)  ② Light activity (Office work, Technician, Housewife with short house work, similar job)  ③ General activity (Housewife with much house work, Manufacturing industry, retail business, teacher)  ④ High activity (Farming, Fishery, Construction industry, similar job)  ⑤ Severe activity (Athlete, Transportation of wood, farmer in busy farming season, similar job)  **2. In recent a week, how many days that body condition is severe tired or do the work out with breath hard more than 10 minutes?**  ① Never (go to #3) ② 1~2 in a week ③ 3~4 in a week ④ 5~6 in a week ⑤ everyday  **3. In recent a week, how many days do the work out with light breathe hard more than 10 minutes?**  ① Never (go to #4) ② 1~2 in a week ③ 3~4 in a week ④ 5~6 in a week ⑤ everyday  **3-1.How long did you do this kind of light breathe hard work out in a day?**  A day ( ) hour ( ) minutes.  **4. In recent a week, how many days did you walk at least more than 10 minutes?**  ① Never ② 1~2 in a week ③ 3~4 in a week ④ 5~6 in a week ⑤ everyday  **4-1. Among this day, how long did you work per day?**  A day ( ) hour ( ) minutes.  **5. Are you smoker?**  ① Every day ( )/per day ② Sometimes ( )/per day ③ Smoker in the past but not now ( duration : ) ④ Never |
| --- |

**Anthropometric measurements**

| **No.** | **Survey Subject** | **Height (cm)** | **Weight (kg)** | **Waist circumference (cm)** | **Hip circumference (cm)** | **Blood pressure**  **(mm/Hg)** |
| --- | --- | --- | --- | --- | --- | --- |
|  |  |  |  |  |  |  |
|  |  |  |  |  |  |  |
|  |  |  |  |  |  |  |
|  |  |  |  |  |  |  |
|  |  |  |  |  |  |  |
|  |  |  |  |  |  |  |
|  |  |  |  |  |  |  |
|  |  |  |  |  |  |  |
|  |  |  |  |  |  |  |
|  |  |  |  |  |  |  |
|  |  |  |  |  |  |  |
|  |  |  |  |  |  |  |
|  |  |  |  |  |  |  |
|  |  |  |  |  |  |  |
|  |  |  |  |  |  |  |
|  |  |  |  |  |  |  |
|  |  |  |  |  |  |  |
|  |  |  |  |  |  |  |
|  |  |  |  |  |  |  |
|  |  |  |  |  |  |  |
|  |  |  |  |  |  |  |
|  |  |  |  |  |  |  |
|  |  |  |  |  |  |  |
|  |  |  |  |  |  |  |
|  |  |  |  |  |  |  |
|  |  |  |  |  |  |  |

**.**

**Eating Behavior Survey**

**※ This survey is about daily life and body activities**

| Eating Behavior Questionnaire | | | Not at all | | | Slightly | | Moderately | | | | Very | | Extremely | |
| --- | --- | --- | --- | --- | --- | --- | --- | --- | --- | --- | --- | --- | --- | --- | --- |
| 1. I eat the soup of noodles thoroughly. | | |  | | |  | |  | | | |  | |  | |
| 2 I eat often (e.g ramen, fish cake, canned food). | | |  | | |  | |  | | | |  | |  | |
| 3. I eat out or deliver food often (2-3 times per week). | | |  | | |  | |  | | | |  | |  | |
| 4. I often eat fish or fish with salt. | | |  | | |  | |  | | | |  | |  | |
| 5. I eat instant food often (e.g hamburger, pizza, etc...). | | |  | | |  | |  | | | |  | |  | |
| 6. I eat potato chips, crackers and cookies often as a snack. | | |  | | |  | |  | | | |  | |  | |
| 7. I habitually add salt or soy sauce to food before I eat. | | |  | | |  | |  | | | |  | |  | |
| 8. I use low salt-product (low-salt soy paste, low-salt soy sauce) | | |  | | |  | |  | | | |  | |  | |
| 9. I ask to cook blandly when I eat out. | | |  | | |  | |  | | | |  | |  | |
| 10. I often eat stewed food or stir-fried food as a side dish. | | |  | | |  | |  | | | |  | |  | |
| 11. I often buy seasoned meat, seafood, and side dish for sale. | | |  | | |  | |  | | | |  | |  | |
| 12. I eat Korean food | | |  | | |  | |  | | | |  | |  | |
| 13. I eat a lot of vegetables every meal. | | |  | | |  | |  | | | |  | |  | |
| 14. I often eat food made of potato, bean, and tofu. | | |  | | |  | |  | | | |  | |  | |
| 15. I eat fruits everyday. | | |  | | |  | |  | | | |  | |  | |
|  | | | Not at all | | | Do not often do that | | Normal | | | | Like | | Strongly like | |
| 16. Do you add a lot of soy sauce or red pepper paste to sliced raw fish, jeon (Korean pancake), or fried food? | | |  | | |  | |  | | | |  | |  | |
|  | | | Not at all | | | Do not often do that | | Normal | | | | Usually confirm | | Always confirm | |
| 17. Do you check the sodium content when you buy processed foods? | | |  | | |  | |  | | | |  | |  | |
| Items (Frequency of food consumption for a last year) | | | Day | | | | | | | Week | | | Month | | |
|  |  |  | 3 | | 2 | | 1 | | | 3~6 | 1~2 | | 1~3 | | 1/month or never |
| 18. How often do you eat Kimchi? | | |  | |  | |  | | |  |  | |  | |  |
| 19. How often do you eat Korean soup or stew? | | |  | |  | |  | | |  |  | |  | |  |
| 20. How often do you eat nuts for a snack? | | |  | |  | |  | | |  |  | |  | |  |
| **Eat out frequency for meal substitute (including delivery food)** | ① barely  ④ 4-5 times per week | ② 1-2 times per week  ⑤ 1 time per day | | | | | | | ③. 3-4 times per week  ⑥ more than 2 times per day | | | | | | |
| **The most frequently eaten food when eating out(including delivery food)**  **(pick 3)** | ① staff restaurant(group meal)  ③ Stew  ⑤ Homemade rice(Rice and side dish)  ⑦ fried rice, rice bowls, simmered fish..  ⑨ Meat | | | ② chinese food  ④ Snack (Dduckbok-gi, ramen..etc)  ⑥ sandwich, toast  ⑧ hamburger, pizza, pasta, etc | | | | | | | | | | | |
| **Considerations when eating out**  **(pick 1)** | ① taste  ④ low-salted | ② health  ⑤ sanitary | | | | | | | ③ cost  ⑥ preparation time  (delivery time) | | | | | | |

**Food frequency questionaries**

**※ Please answer how often you have had the following food or food prepared with the main ingredients for the last 6 months.**

| **Dish** | | | **Size** | **Serving size** | | | **Frequency** | | | | | | | | |
| --- | --- | --- | --- | --- | --- | --- | --- | --- | --- | --- | --- | --- | --- | --- | --- |
|  |  |  |  |  |  |  | **Day** | | | **Week** | | | **Month** | | **Never** |
|  |  |  |  | **more** | **mormal** | **less** | **3** | **2** | **1** | **5-6** | **3-4** | **1-2** | **2-3** | **1** |  |
| **Rice** | **1,** | **Kimchi fried rice** | **1plate(160g)** |  |  |  |  |  |  |  |  |  |  |  |  |
|  | **2** | **Gimbap** | **1roll(140g)** |  |  |  |  |  |  |  |  |  |  |  |  |
|  | **3** | **Bibimbap** | **1bowl(240g)** |  |  |  |  |  |  |  |  |  |  |  |  |
|  | **4** | **Curry rice** | **1bowl(230g)** |  |  |  |  |  |  |  |  |  |  |  |  |
|  | **5** | **Bowl of rice served with toppings** | **1bowl(200g)** |  |  |  |  |  |  |  |  |  |  |  |  |
|  | **6** | **(Rice) porridge** | **1bowl(80g)** |  |  |  |  |  |  |  |  |  |  |  |  |
| **Noodles** | **7** | **Ramen** | **1bowl(140g)** |  |  |  |  |  |  |  |  |  |  |  |  |
|  | **8** | **Chopped noodles (noodle soup)** | **1bowl(170g)** |  |  |  |  |  |  |  |  |  |  |  |  |
|  | **9** | **Chopped noodles (noodle soup) + sauce** | **1bowl(170g) +sauce(15g)** |  |  |  |  |  |  |  |  |  |  |  |  |
|  | **10** | **Spicy noodles** | **1bowl(170g)** |  |  |  |  |  |  |  |  |  |  |  |  |
|  | **11,** | **Banquet noodles** | **1bowl(190g)** |  |  |  |  |  |  |  |  |  |  |  |  |
|  | **12** | **Banquet noodles + sauce** | **1bowl(170g) +sauce(15g)** |  |  |  |  |  |  |  |  |  |  |  |  |
|  | **13** | **Buckwheat noodles** | **1bowl(190g)** |  |  |  |  |  |  |  |  |  |  |  |  |
|  | **14** | **Cold buckwheat noodles** | **1bowl(160g)** |  |  |  |  |  |  |  |  |  |  |  |  |
|  | **15** | **Spicy buckwheat noodles** | **1bowl(200g)** |  |  |  |  |  |  |  |  |  |  |  |  |
|  | **16** | **Udon (noodles) or Jjamppong (chinese-style noodles with vegetables and seafood)** | **1bowl(150g)** |  |  |  |  |  |  |  |  |  |  |  |  |
|  | **17** | **Jajangmyeon (noodles with black soybean sauce)** | **1bowl(110g)** |  |  |  |  |  |  |  |  |  |  |  |  |
| **Bread** | **18** | **Sandwich** | **1개(130g)** |  |  |  |  |  |  |  |  |  |  |  |  |
|  | **19** | **Pizza** | **1piece(150g)** |  |  |  |  |  |  |  |  |  |  |  |  |
| **Soup, stew** | **20** | **Soybean paste soup** | **1bowl(100g)** |  |  |  |  |  |  |  |  |  |  |  |  |
|  | **21** | **Seaweed soup** | **1bowl(32g)** |  |  |  |  |  |  |  |  |  |  |  |  |
|  | **22** | **Kimchi stew** | **1bowl(40g)** |  |  |  |  |  |  |  |  |  |  |  |  |
|  | **23** | **Bean sprout soup** | **1bowl(40g)** |  |  |  |  |  |  |  |  |  |  |  |  |
|  | **24** | **Beef soup** | **1bowl(70g)** |  |  |  |  |  |  |  |  |  |  |  |  |
|  | **25** | **Soybean paste stew** | **1bowl(100g)** |  |  |  |  |  |  |  |  |  |  |  |  |
|  | **26** | **Kimchi stew** | **1bowl(120g)** |  |  |  |  |  |  |  |  |  |  |  |  |
|  | **27** | **Fish stew** | **1bowl(130g)** |  |  |  |  |  |  |  |  |  |  |  |  |
|  | **28** | **Tofu stew** | **1bowl(180g)** |  |  |  |  |  |  |  |  |  |  |  |  |

| **Dish** | | | **Size** | **Serving size** | | | **Frequency** | | | | | | | | |
| --- | --- | --- | --- | --- | --- | --- | --- | --- | --- | --- | --- | --- | --- | --- | --- |
|  |  |  |  |  |  |  | **Day** | | | **Week** | | | **Month** | | **Never** |
|  |  |  |  | **more** | **mormal** | **less** | **3** | **2** | **1** | **5-6** | **3-4** | **1-2** | **2-3** | **1** |  |
| **Soup, stew** | **29** | **Canned fish stew** | **1bowl(120g)** |  |  |  |  |  |  |  |  |  |  |  |  |
|  | **30** | **Squid stew** | **1bowl(140g)** |  |  |  |  |  |  |  |  |  |  |  |  |
|  | **31** | **Pork back-bone stew** | **1bowl(110g)** |  |  |  |  |  |  |  |  |  |  |  |  |
|  | **32** | **Beef-bone stew** | **1bowl(90g)** |  |  |  |  |  |  |  |  |  |  |  |  |
|  | **33** | **Spicy beef soup**  **(yukgaejang)** | **1bowl(160g)** |  |  |  |  |  |  |  |  |  |  |  |  |
|  | **34** | **Spicy seafood stew (Haemul-tang)** | **1bowl(110g)** |  |  |  |  |  |  |  |  |  |  |  |  |
|  | **35** | **Fish cake soup (Eomuk tang)** | **1bowl(130g)** |  |  |  |  |  |  |  |  |  |  |  |  |
| **Fish and shellfish** | **36** | **Grilled fish** | **1cut(60g)** |  |  |  |  |  |  |  |  |  |  |  |  |
|  | **37** | **Stir-fried anchovies** | **1small dish(30g)** |  |  |  |  |  |  |  |  |  |  |  |  |
|  | **38** | **Stir-fried boiled fish paste** | **1plate(155g)** |  |  |  |  |  |  |  |  |  |  |  |  |
|  | **39** | **Stir-fried dried squid** | **1plate(30g)** |  |  |  |  |  |  |  |  |  |  |  |  |
|  | **40** | **Salted dry fish** | **1plate(110g)** |  |  |  |  |  |  |  |  |  |  |  |  |
|  | **41** | **Pan-fried fish fillet** | **1cut(70g)** |  |  |  |  |  |  |  |  |  |  |  |  |
|  | **42** | **Pan-fried fish fillet** | **1piece(100g)** |  |  |  |  |  |  |  |  |  |  |  |  |
|  | **43** | **Pan-fried fish fillet**  **+ Soy sauce (or mixed with vinegar)** | **1piece(100g)**  **+Soy sauce(15g)** |  |  |  |  |  |  |  |  |  |  |  |  |
|  | **44** | **Seafood pancake** | **1piece(110g)** |  |  |  |  |  |  |  |  |  |  |  |  |
|  | **45** | **Stir-fried squid or small octopus** | **1plate(120g)** |  |  |  |  |  |  |  |  |  |  |  |  |
| **Meat, egg, and pulse** | **46** | **steamed eggs** | **1small bowl(50g)** |  |  |  |  |  |  |  |  |  |  |  |  |
|  | **47** | **Stir-fried pork** | **1plate(100g)** |  |  |  |  |  |  |  |  |  |  |  |  |
|  | **48** | **Slices of boiled meat (Napa wraps with pork)** | **1plate(120g)** |  |  |  |  |  |  |  |  |  |  |  |  |
|  | **49** | **Braised tofu** | **3pieces(60g)** |  |  |  |  |  |  |  |  |  |  |  |  |
|  | **50** | **Korean sausage (sundae)** | **1plate(90g)** |  |  |  |  |  |  |  |  |  |  |  |  |
|  | **51** | **Korean sausage (sundae) + salt** | **1plate(90g) + salt(1g)** |  |  |  |  |  |  |  |  |  |  |  |  |
|  | **52** | **Korean sausage (sundae) + soybean paste** | **1plate(90g)**  **+ soybean paste(20g)** |  |  |  |  |  |  |  |  |  |  |  |  |
|  | **53** | **Pork cutlet** | **2pieces(100g)** |  |  |  |  |  |  |  |  |  |  |  |  |
|  | **54** | **Pork cutlet + sauce** | **2pieces(100g)+sauce(20g)** |  |  |  |  |  |  |  |  |  |  |  |  |
|  | **55** | **Stewed chicken** | **1plate(120g)** |  |  |  |  |  |  |  |  |  |  |  |  |
|  | **56** | **Braised spicy chicken** | **1plate(120g)** |  |  |  |  |  |  |  |  |  |  |  |  |
|  | **57** | **Pork ribs** | **4pieces(100g)** |  |  |  |  |  |  |  |  |  |  |  |  |
|  | **58** | **Pork belly**  **+ Salt** | **3pieces(50g)**  **+ Salt(14g)** |  |  |  |  |  |  |  |  |  |  |  |  |
|  | **59** | **Pork belly**  **+ Bean paste (Ssamjang)** | **3pieces(50g)**  **+ Bean paste (Ssamjang)(20g)** |  |  |  |  |  |  |  |  |  |  |  |  |

| **Dish** | | | **Size** | **Serving size** | | | **Frequency** | | | | | | | | |
| --- | --- | --- | --- | --- | --- | --- | --- | --- | --- | --- | --- | --- | --- | --- | --- |
|  |  |  |  |  |  |  | **Day** | | | **Week** | | | **Month** | | **Never** |
|  |  |  |  | **more** | **mormal** | **less** | **3** | **2** | **1** | **5-6** | **3-4** | **1-2** | **2-3** | **1** |  |
| **Meat, egg, and pulse** | **60** | **Bulgogi (sliced and seasoned, barbequed beef)** | **1plate(100g)** |  |  |  |  |  |  |  |  |  |  |  |  |
|  | **61** | **Fried chicken** | **2pieces(70g)** |  |  |  |  |  |  |  |  |  |  |  |  |
| **Seasoned vegetables, raw vegetables (salad)** | **62** | **Grilled seaweed** | **8pieces(4g)** |  |  |  |  |  |  |  |  |  |  |  |  |
|  | **63** | **Wrapped rice + Bean paste (Ssamjang)** | **Bean paste (Ssamjang)(25g)** |  |  |  |  |  |  |  |  |  |  |  |  |
|  | **64** | **Seasoned spinach** | **1plate(70g)** |  |  |  |  |  |  |  |  |  |  |  |  |
|  | **65** | **Vegetable pancake** | **1piece(70g)** |  |  |  |  |  |  |  |  |  |  |  |  |
|  | **66** | **Vegetable pancake + Soy Sauce** | **1piece(70g)**  **+ Soy Sauce(15g)** |  |  |  |  |  |  |  |  |  |  |  |  |
| **Kimchi** | **67** | **Kimchi** | **1small bowl(40g)** |  |  |  |  |  |  |  |  |  |  |  |  |
|  | **68** | **(Small radish) Kimchi** | **1small bowl(50g)** |  |  |  |  |  |  |  |  |  |  |  |  |
|  | **69** | **(Young leafy radish) Kimchi** | **1small bowl(40g)** |  |  |  |  |  |  |  |  |  |  |  |  |
|  | **70** | **Watery kimchi** | **1bowl(80g)** |  |  |  |  |  |  |  |  |  |  |  |  |
|  | **71** | **Stir-fried kimchi** | **1plate(160g)** |  |  |  |  |  |  |  |  |  |  |  |  |
|  | **72** | **Kimchi pancake** | **1piece(100g)** |  |  |  |  |  |  |  |  |  |  |  |  |
| **Pickled vegetabl es** | **73** | **Stuffed cucumber pickles** | **2pieces(70g)** |  |  |  |  |  |  |  |  |  |  |  |  |
|  | **74** | **Pickled garlic** | **1small dish(20g)** |  |  |  |  |  |  |  |  |  |  |  |  |
|  | **75** | **Onion pickles** | **1small dish(20g)** |  |  |  |  |  |  |  |  |  |  |  |  |
|  | **76** | **Fermented squid** | **1small dish(20g)** |  |  |  |  |  |  |  |  |  |  |  |  |
| **Spicery** | **77** | **(Fried) + (tomato) ketchup** | **1Ts(16g)** |  |  |  |  |  |  |  |  |  |  |  |  |
|  | **78** | **Table salt** | **1Ts(8g)** |  |  |  |  |  |  |  |  |  |  |  |  |

**• Age : years · • Gender :** □ Male □ Female

**Do you like salty food?**

① Strongly dislike

② Dislike

③ Neutral

④ Like

⑤ Strongly like

**Do you think you eat salty food?**

① Usually bland

② Usually a little bland

③ Regular salt (especially prefer not eating salty or bland food)

④ A little salty

⑤ Very salty

| **Education**  **(graduate)** | ① Elementary school  ④ College | ② Middle school  ⑤ Graduate school or higher | ③ High school |
| --- | --- | --- | --- |
| **Occupation** | ① Specialized job  ④ housewife | ② retail business/service industry /self-employed  ⑤ company employee | ③ government employee  ⑥ University students / others |
| **Household income (ten thousand won)** | ① ≤ 100  ④ 300~400 | ② 100~200  ⑤ 400~500 | ③ 200~300  ⑥ ≥ 500 |

**24-hour recall questionnaire (day 1)**

**1 Please tell me what you eat and how much you have eaten during the day (including dietary supplements)**

| Meal classification | Time of day | Place | Menu or foods | Serving size | | | Name of preparation | Material amount | | | Remarks | |
| --- | --- | --- | --- | --- | --- | --- | --- | --- | --- | --- | --- | --- |
|  |  |  |  | Eyeball | Volume | Wegiht |  | Eyeball | Volume | Wegiht | Band name | Manufacturer |
|  |  |  |  |  |  |  |  |  |  |  |  |  |
|  |  |  |  |  |  |  |  |  |  |  |  |  |
|  |  |  |  |  |  |  |  |  |  |  |  |  |
|  |  |  |  |  |  |  |  |  |  |  |  |  |
|  |  |  |  |  |  |  |  |  |  |  |  |  |
|  |  |  |  |  |  |  |  |  |  |  |  |  |
|  |  |  |  |  |  |  |  |  |  |  |  |  |
|  |  |  |  |  |  |  |  |  |  |  |  |  |
|  |  |  |  |  |  |  |  |  |  |  |  |  |
|  |  |  |  |  |  |  |  |  |  |  |  |  |
|  |  |  |  |  |  |  |  |  |  |  |  |  |
|  |  |  |  |  |  |  |  |  |  |  |  |  |
|  |  |  |  |  |  |  |  |  |  |  |  |  |
|  |  |  |  |  |  |  |  |  |  |  |  |  |
|  |  |  |  |  |  |  |  |  |  |  |  |  |
|  |  |  |  |  |  |  |  |  |  |  |  |  |
|  |  |  |  |  |  |  |  |  |  |  |  |  |
|  |  |  |  |  |  |  |  |  |  |  |  |  |
|  |  |  |  |  |  |  |  |  |  |  |  |  |
|  |  |  |  |  |  |  |  |  |  |  |  |  |

**2 How much is the amount of food consumed yesterday compared to the usual diet?**

① It consumed more than usual ② It was similar to usual ③ It consumed less than usual

**3 How much water do you consume in a day [water and barley tea etc]?** ( ) Cup (200ml)

**24-hour recall questionnaire (day 5)**

**1 Please tell me what you eat and how much you have eaten during the day (including dietary supplements)**

| Meal classification | Time of day | Place | Menu or foods | Serving size | | | Name of preparation | Material amount | | | Remarks | |
| --- | --- | --- | --- | --- | --- | --- | --- | --- | --- | --- | --- | --- |
|  |  |  |  | Eyeball | Volume | Wegiht |  | Eyeball | Volume | Wegiht | Band name | Manufacturer |
|  |  |  |  |  |  |  |  |  |  |  |  |  |
|  |  |  |  |  |  |  |  |  |  |  |  |  |
|  |  |  |  |  |  |  |  |  |  |  |  |  |
|  |  |  |  |  |  |  |  |  |  |  |  |  |
|  |  |  |  |  |  |  |  |  |  |  |  |  |
|  |  |  |  |  |  |  |  |  |  |  |  |  |
|  |  |  |  |  |  |  |  |  |  |  |  |  |
|  |  |  |  |  |  |  |  |  |  |  |  |  |
|  |  |  |  |  |  |  |  |  |  |  |  |  |
|  |  |  |  |  |  |  |  |  |  |  |  |  |
|  |  |  |  |  |  |  |  |  |  |  |  |  |
|  |  |  |  |  |  |  |  |  |  |  |  |  |
|  |  |  |  |  |  |  |  |  |  |  |  |  |
|  |  |  |  |  |  |  |  |  |  |  |  |  |
|  |  |  |  |  |  |  |  |  |  |  |  |  |
|  |  |  |  |  |  |  |  |  |  |  |  |  |
|  |  |  |  |  |  |  |  |  |  |  |  |  |
|  |  |  |  |  |  |  |  |  |  |  |  |  |
|  |  |  |  |  |  |  |  |  |  |  |  |  |
|  |  |  |  |  |  |  |  |  |  |  |  |  |

**2 How much is the amount of food consumed yesterday compared to the usual diet?**

① It consumed more than usual ② It was similar to usual ③ It consumed less than usual

**3 How much water do you consume in a day [water and barley tea etc]?** ( ) Cup (200ml)
